# Explicit Performance in Girls and Implicit Processing in Boys: A Simultaneous fNIRS–ERP Study on Second Language Syntactic Learning in Young Adolescents

**DOI:** 10.3389/fnhum.2018.00062

**Published:** 2018-03-08

**Authors:** Lisa Sugiura, Masahiro Hata, Hiroko Matsuba-Kurita, Minako Uga, Daisuke Tsuzuki, Ippeita Dan, Hiroko Hagiwara, Fumitaka Homae

**Affiliations:** ^1^Department of Language Sciences, Graduate School of Humanities, Tokyo Metropolitan University, Tokyo, Japan; ^2^Research Center for Language, Brain and Genetics, Tokyo Metropolitan University, Tokyo, Japan; ^3^Applied Cognitive Neuroscience Lab, Faculty of Science and Engineering, Chuo University, Tokyo, Japan; ^4^Department of Welfare and Psychology, Health Science University, Yamanashi, Japan

**Keywords:** proficiency, sex differences, working memory, sentence, phrase structure, syntax, adaptive hemodynamic response function, reading span test

## Abstract

Learning a second language (L2) proceeds with individual approaches to proficiency in the language. Individual differences including sex, as well as working memory (WM) function appear to have strong effects on behavioral performance and cortical responses in L2 processing. Thus, by considering sex and WM capacity, we examined neural responses during L2 sentence processing as a function of L2 proficiency in young adolescents. In behavioral tests, girls significantly outperformed boys in L2 tests assessing proficiency and grammatical knowledge, and in a reading span test (RST) assessing WM capacity. Girls, but not boys, showed significant correlations between L2 tests and RST scores. Using functional near-infrared spectroscopy (fNIRS) and event-related potential (ERP) simultaneously, we measured cortical responses while participants listened to syntactically correct and incorrect sentences. ERP data revealed a grammaticality effect only in boys in the early time window (100–300 ms), implicated in phrase structure processing. In fNIRS data, while boys had significantly increased activation in the left prefrontal region implicated in syntactic processing, girls had increased activation in the posterior language-related region involved in phonology, semantics, and sentence processing with proficiency. Presumably, boys implicitly focused on rule-based syntactic processing, whereas girls made full use of linguistic knowledge and WM function. The present results provide important fundamental data for learning and teaching in L2 education.

## Introduction

Language and communication have long been a focus of attention in multiple disciplines, such as philosophy, cognitive science, neuroscience, and information sciences, because of its central role in human activity. The fluent use of not only a first language (L1), but also a second language (L2) is extremely important for communication between people with different linguistic and cultural backgrounds, especially given the rapid globalization in various economic and social fields.

It is widely accepted that if exposed early enough, any normally developing child from any part of the world is capable of acquiring his/her native language in a short period of time with little or no explicit instruction. Based on Chomsky’s proposition, language is acquired through the use of an innate language acquisition device (Chomsky, 1965). Also, the functional properties of L1 develop along a typical maturational path, suggesting a universal genetic basis for language acquisition. While it may be well understood that individual abilities (e.g., intelligence, aptitude), states (e.g., motivation, attitudes), traits (e.g., extroversion, introversion) and such have little, if any, effect on L1 acquisition, we have seen that individual differences do affect L2 development. Similar aspects of developmental sequences in L1 and L2 have been reported (Dulay and Burt, 1974; Hatch and Wagner-Gough, 1976), but L2 learning rate and ultimate attainment are quite variable. Also, many factors, including the factors mentioned above, seem to have effects on variances in L2 proficiency with functional changes in the brain in some cases; we focused on the L2 proficiency considering the effect of sex and working memory (WM) capacity in the present study, on the basis of the following background.

Previous neuroimaging studies investigating language processing have shown that distinctive brain regions are activated in response to different linguistic components of language comprehension and production, such as phonology (word sound), semantics (meaning), and syntax (sentence structure). Also, accumulated data have demonstrated that, broadly, analogous brain areas are recruited in L1 and L2 (e.g., Rüschemeyer et al., 2006; Suh et al., 2007; for reviews, see Abutalebi, 2008; Kotz, 2009). However, the degree of activation, activation latency, and/or precise regions activated vary as a function of proficiency (Tatsuno and Sakai, 2005; Golestani et al., 2006; for a review, see Kotz, 2009). These differences in brain response to linguistic stimuli are pronounced at the beginning of L2 learning and/or when L2 is processed with a non-native-like proficiency (for reviews, see Abutalebi, 2008; Kotz, 2009).

Although it may be understood that individual differences have little effect on L1 acquisition, previous behavioral studies on L1 acquisition have reported sex differences, in which faster language development in girls is consistently found ranging from word level (Doran, 1907; Huttenlocher et al., 1991; Bauer et al., 2002) to phrase and sentence level (Nelson, 1973; Murray et al., 1990; Roulstone et al., 2002). A number of neuroimaging studies also identified sex differences (word level: Shaywitz et al., 1995; Pugh et al., 1996; Baxter et al., 2003; Clements et al., 2006; Plante et al., 2006; Burman et al., 2008, 2013; sentence level: Kansaku et al., 2000; Phillips et al., 2001), although controversy remains (Frost et al., 1999; Weiss et al., 2003; Sommer et al., 2004, 2008). The existence of sex differences seems to be task-dependent: while the studies that identified sex differences employed passive listening tasks (Kansaku et al., 2000; Phillips et al., 2001) and phonological tasks (Shaywitz et al., 1995; Pugh et al., 1996; Clements et al., 2006), the studies that did not find sex differences utilized language comprehension tasks and verbal fluency tasks (Frost et al., 1999; Weiss et al., 2003; Wallentin, 2009).

Adolescence is a period of L2 learning in school, and of increased divergence between the sexes in both physical and behavioral characteristics (Sisk and Zehr, 2005; Paus, 2010); however, sex differences in neural plasticity and development in L2 learning during this period remain poorly understood. In our previous study, we investigated how L2 proficiency changes cortical response during word processing in elementary school children and found significant sex differences in cortical response in relation to L2 proficiency (Sugiura et al., 2015). Therefore, in the present study with junior high school students, we further explored how the L2 proficiency of the learners affects cortical response during sentence-level syntactic processing, which is the major focus of previous language processing research (Cambria and White, 2014), and the analyses were carried out considering sex as a possible factor. Sentence processing requires a great deal of syntactic knowledge and computation relative to lexical/word processing, making it better suited for investigating correlations between L2 proficiency at the sentence level and cortical response.

In addition to sex, we also considered WM capacity in examining the relationship between L2 proficiency and behavioral performance, as well as that between L2 proficiency and cortical response during L2 sentence processing. This is because the role of WM in syntactic processing (King and Just, 1991), as well as L2 proficiency (Miyake and Friedman, 1998) has been reported. WM is the ability to retain information during short periods of time while simultaneously processing both old and new information. King and Just (1991) conducted experiments with 94 college students and showed that individual differences in syntactic processing are governed in part by the amount of WM capacity available for language comprehension processes. Miyake and Friedman (1998) posited that WM for language may be one central component of language aptitude, and play a role in individual differences in L2 proficiency among adult learners. Also they suggested that the role of WM in the performance of linguistic tasks may be stronger in L2 than in L1.

Although influences of verbal WM on sentence processing in adults have been reported as above, there is little literature about such influences in normally developing adolescents. Lehto (1995) investigated the relationship between WM capacity and school achievement in adolescents (15–16 years), and reported that WM capacity had a highly significant correlation with both foreign and native language performance, and suggested that the phonological loop is specifically related to foreign language learning. However, until now, the relationship between L2 proficiency and cortical responses during L2 sentence processing in adolescents, taking WM capacity and sex into consideration, have not been investigated. It should be noted that the idea of sex differences in the behavioral performance of WM tasks is controversial: while some studies have reported a female advantage for verbal WM (Kramer et al., 1997; Speck et al., 2000; Pauls et al., 2013), others have found that there are no significant sex differences during such tasks (Goldstein et al., 2005; Nagel et al., 2007). Interestingly, regardless of behavioral performance, sex differences in cortical activations have been observed; for example, females have exhibited cortical activation in left-sided dominance (Speck et al., 2000) and greater activation in the middle, inferior, and orbital prefrontal cortices (Goldstein et al., 2005) compared to males. The authors of these studies discussed that the differences in cortical activation resulted from different strategies between sexes.

In the present study, we conducted simultaneous functional near-infrared spectroscopy (fNIRS) and event-related potential (ERP) measurements to assess cortical responses during L2 sentence processing. The usefulness and advantages of simultaneous fNIRS–ERP measurements in language studies have been reported (Horovitz and Gore, 2004; for a review, see Wallois et al., 2012); however, not many studies have yet made use of this method, especially those dealing with children and adolescents. By integrating fNIRS and ERP data, we benefit from both the spatial resolution of fNIRS and the high temporal resolution of ERP. While fNIRS can detect global cortical activation during syntactic processing, ERP is expected to provide more precise information about the timing of language processing after the target (violation/ungrammatical) point in a sentence.

fNIRS has been applied to language studies of newborns, infants, children, and adults in both healthy populations and in patients with neurological and psychiatric disorders (Quaresima et al., 2012; Homae, 2014). Considering previous literature for both L1 and L2 (Kovelman et al., 2008; Oi et al., 2010; Minagawa-Kawai et al., 2011; Sugiura et al., 2011, 2015), we hypothesized that late adolescent L2 learners have greater activation in the left relative to the right temporal and frontal language areas (Wernicke’s area and Broca’s area) during sentence-level processing as L2 proficiency increases, but since few studies have aimed at understanding sex differences as well as the involvement of general cognitive functions (other than language-specific functions) such as WM for late L2 learners, we explored these questions in the present study.

In ERP research, four main components have been reported for language processing: early left anterior negativity (ELAN), left anterior negativity (LAN), N400, and P600, and among these, ELAN, LAN, and P600 are considered to be indices of syntactic processing. The ELAN component, often lateralized to the left hemisphere, occurs in the latency range of 100–300 ms, is assumed to reflect automatic syntactic-structure building (Hahne and Friederici, 1999), and is often seen in response to phrase structure violations (Friederici, 1995). The LAN component, again often left-lateralized, occurs in the range of 300–500 ms. It also reflects syntactic processing and appears to correlate particularly well with morphosyntactic and thematic processes (Friederici, 2002). The P600 component has been interpreted as reflecting syntactic reanalysis/repair (Friederici et al., 1999, 2002) in language comprehension, while the N400 component is known to be a normal response, reflecting semantic-related, but not syntactic, processes (Kutas and Hillyard, 1980; Kutas and Federmeier, 2000).

We employed passive listening as our experimental condition during brain measurements, since listening comprehension of verbal auditory stimuli is one of the most important and fundamental of the four skills (listening, speaking, reading, and writing) in language learning as it is the skill most often used in everyday life. During passive listening, participants heard syntactically correct and incorrect sentences. For ERP analyses, we focused on differences in the time courses of neural activation for syntactic processing between boys and girls. For fNIRS analyses, cortical responses and representation during syntactic sentence processing were examined as a function of L2 proficiency, considering sex and WM capacity.

## Materials and Methods

### Participants

Participants of this study initially included 58 normally developing Japanese junior high school students in Tokyo. All participants completed a questionnaire before commencing this study. Each of the participants and their parents gave written informed consent before his or her participation in this study. All of the procedures in this study were approved by the Human Subject Ethics Committee of Tokyo Metropolitan University. The Edinburgh Handedness Inventory (Oldfield, 1971) was used to determine hand dominance. Participants who participated in this study are all right-handed, no participants had psychiatric disorders, and the L1 of all participants and their parents was Japanese. As English had not been introduced as a mandatory academic subject at the elementary school level in Japan at the time the data were collected, participants’ age of exposure to L2 was 12–13 years old. Although participants’ L1 proficiency was not examined, it can be assumed that our participants had relatively equal L1 proficiency for the following reasons. The present study did not include individuals with one or more parents whose native language was other than Japanese (including native English speakers), so that participants’ daily language use was limited to Japanese. All the academic subjects taught in the schools attended by the participants were taught in L1, and their common, everyday language at their schools was Japanese. Also, Japan requires 9 years of compulsory education, 6 at elementary school, and 3 at junior high school. Since that education is compulsory and relatively uniform throughout the country, a relative equality of educational outcomes (including L1 proficiency in daily use) is seen in Japan. Thus, no participants were excluded at this stage. Also, no participants in this study had experience living abroad or in any English-speaking environment, or of attending international school or bilingual/immersion school. However, five participants were excluded from the analyses because of poor data quality caused by insufficient contact between the optodes and scalp (three participants) or data corruption due to deficient event triggers (two participants) in the fNIRS measurements. Consequently, 53 participants (31 boys and 22 girls, aged 12–15 years, mean age = 13.88, standard deviation of age = 0.93) were used for the current analyses. A *t*-test confirmed that there was no significant age difference between sexes [*t*(51) = -1.079, *P* = 0.279, n.s.].

Note that in the field of L2 acquisition/learning, there are generally two types of acquisition/learning environments. One is an environment where the target language is not typically spoken in everyday life and is generally acquired through formal instruction in a classroom setting after a native language has been acquired. In this case, the term “foreign language (FL)” is often used. Another is a more natural environment similar to that of native language acquisition. In this case, the term “L2” is usually used. However, L2 is often used more broadly to refer to the acquisition/learning of a language other than the native language. Therefore, in the present study, the broader definition of L2 is used although our participants learned English as an L2 in a classroom environment through formal instruction.

### Behavioral Data Acquisition

We had participants of the same age with different levels of English proficiency, thus in order to assess their overall English language proficiency, the Cambridge basic level English language exam, called the Key English Test (KET), was administered. KET is an elementary level exam focusing on basic everyday communication in written, and spoken English. It is the easiest of the Cambridge English exams, requiring students to have a basic knowledge of English. This qualification demonstrates that students are able to understand very basic instructions, both written and spoken, and the use of simple expressions and phrases. The KET exam consists of reading, writing, listening, and speaking sections; however, the speaking section was not used in the present study.

After the simultaneous fNIRS–ERP measurements, the students also took grammar tests, which used the same sentences as those presented aurally during the measurements. The grammar tests consisted of both listening and reading versions in order to assess the students’ grammatical knowledge of the English language, regardless of sensory modality (auditory or visual). Details of the grammar tests are provided in the Section “Experimental Conditions.” In all, four English language tests were administered to the junior high school students: KET listening test (KET_L), KET reading and writing test (KET_RW), grammar listening test (Grammar_L), and grammar reading test (Grammar_R).

We also examined whether auditory L2 performance correlated with WM capacity using a reading span test (RST), which was designed to measure the combined processing and storage capacity of WM during reading (Daneman and Carpenter, 1980). We used a Japanese version of the RST (Osaka and Osaka, 1992). Empirical evidence shows that WM capacity is an excellent predictor of performance on a variety of complex cognitive tasks, including tasks that measure language comprehension ability (Daneman and Merikle, 1996). The procedure and materials of the RST used in this study are briefly described below. Participants were presented with sentences typed on a card, and were instructed to read them aloud from individual cards while remembering target words underlined in red (one target word per sentence). The sentences presented were at the lower elementary school level so that they were easy enough for the adolescent participants to read. At the end of each set, they were presented with a blank cue card, at which time they were asked to orally recall the red underlined words from each sentence. There were four span levels varying from two sentences per set to five sentences per set; at each span level, five sets were prepared. The test was carried out in order of difficulty, beginning with two sentences per set and progressing to five sentences per set. Participants were given 5 s per word to orally recall the words (e.g., 10 s for two sentences per set, and 25 s for five sentences per set). The RST score included the total number of correct words recalled, the maximum being 70 (2 × 5 + 3 × 5 + 4 × 5 + 5 × 5).

### Experimental Conditions

Using simultaneous fNIRS–ERP, we measured the participants’ cortical hemodynamic changes and electrophysiological responses as they listened to sentences presented aurally in their L2 (English). While listening, participants viewed muted films to avoid movement artifacts and/or falling asleep while auditory stimuli were presented. The sentences presented include syntactically and semantically correct sentences (correct sentences: NP VP NP PP—NP, noun phrase; VP, verb phrase; and PP, prepositional phrase), ungrammatical sentences with changes in verb–object order (incorrect sentences: NP NP VP PP), and ungrammatical sentences with no VP (filler sentences: NP NP NP PP). Examples of the three types of sentences are given in **Table 1**. There were 48 sentences in each condition, so that a total of 144 sentences (48 sentences × 3 conditions) were presented. In the present study, syntactically correct- and incorrect-sentence conditions were used for data analyses.

**Table 1:** Examples of the three types of sentences presented during the simultaneous fNIRS–ERP measurements and used for grammar tests.

	First phrase	Second phrase	Third phrase	Fourth phrase
Correct	My grandma (NP)	baked (VP)	a cake (NP)	in the afternoon. (PP)
Incorrect	My grandma (NP)	a cake (NP)	baked (VP)	in the afternoon. (PP)
Filler	My grandma (NP)	a cake (NP)	the cookies (NP)	with her aunt. (PP)


During brain measurements, participants sat on a chair in a shielded room. A chin rest was used to maintain the head position. We used an event-related design. The software program Optseq^1^ was used to optimally randomize the order of and spacing between stimuli, which prevents participants from anticipating each stimulus so as to detect brain response to auditory sentence stimuli. In addition to the 144 sentences (48 sentences × 3 conditions), 147 no-sound and 5 pure-tone events were included with the same durations as the sentences presented, which yielded a total of 296 events. One trial was 4000 ms in length and the total time of brain measurements was 1184 s (approximately 19.7 min). Average time for each of the four phrases contained within a sentence was 607, 497, 625, and 1149 ms for the first, second, third, and fourth phrases, respectively.) To ensure that participants were awake and heard the auditory stimuli throughout the brain measurements, participants were required to make simple keypress responses to the pure tone stimuli presented five times, and it was determined in advance that participants who responded less than four out of five times would be excluded from the analyses. Fortunately, as all participants responded at least four times, no participants were excluded.

### Data Acquisition and Analyses

We conducted simultaneous fNIRS–ERP measurements to assess brain responses during L2 syntactic sentence processing. The fNIRS system enables us to measure cortical hemodynamic changes and the ERP system enables us to measure electrophysiological responses.

#### ERP Data Acquisition

The continuous electroencephalograms (EEGs) were recorded using Ag/AgCl electrodes (EASYCAP GmbH, Germany) placed at five positions on the scalp (Fz, Cz, Pz, F5, and F6), which were located according to the international 10–20 electrode system (Jasper, 1958). In addition, electrodes were placed on the left and right ear lobes, and the left earlobe electrode was used as the online reference. Eye movements were monitored using electrooculograms recorded with electrodes placed above the right and below the left outer canthi. The EEGs were amplified with NuAmps (Neuroscan, Charlotte, NC, United States), recorded with a bandpass of 0.1–100 Hz, and digitized with a sampling rate of 500 Hz. Electrode impedance was kept below 5 kΩ.

#### ERP Data Analyses

The same participants for the fNIRS analysis were used in the EEG data analysis. An ocular artifact reduction algorithm implemented in the Neuroscan system (Semlitsch et al., 1986) was applied to the continuous EEGs to reduce the effect of eye blink artifacts. The EEGs were re-referenced to a linked earlobe off-line. The EEG data were filtered with a zero-phase, low-pass filter (30 Hz/12 dB). We focused on the syntactic violation point in the second phrase of each sentence. The averaging epoch was 1200 ms, starting from 200 ms before the onset of the second phrase as a baseline correction and ending at 1000 ms. Trials with amplitudes exceeding ±100 μV were excluded from the analysis as artifacts.

On the basis of the “three-phase model” of language comprehension (Friederici, 2002, 2011), we performed an analysis of variance (ANOVA) in the following time windows: phase 1 (100–300 ms), phase 2-a (300–450 ms), phase 2-b (450–600 ms), and phase 3 (600–800 ms). Phase 2 was divided into two time windows because, although the time window of the major components of this phase (i.e., LAN and N400) is described as between 300 and 500 ms, these components are often observed until 600 ms (e.g., Friederici et al., 2004; Rossi et al., 2005; Hahne et al., 2006). The significance level was 0.05. Greenhouse–Geisser correction was used to correct for violations of sphericity. The original degrees of freedom are used with epsilon (ε) and corrected probability levels.

#### fNIRS Data Acquisition

For fNIRS measurements, we used an fNIRS system (ETG-4000, Hitachi Medical Co., Tokyo, Japan) equipped with a 3 × 5 array of optodes consisting of eight laser diodes and seven light detectors alternately placed at an inter-optode distance of 3 cm, which resulted in a total of 22 channels arranged on each side of the participant’s head. The middle column of the 3 × 5 array was placed along the coronal reference curve (T3-C3-Cz-C4-T4) of the international 10–20 system (Jurcak et al., 2005, 2007), so that the lower edge of the array was placed directly above the ear. The highest sensitivity of hemodynamic changes in the lateral cortical region encompassing a pair of optodes is expected to be localized at the midpoint between the optodes (Okada et al., 1997). This point served as the location of a channel. Optical signals from individual channels were collected at two different wavelengths (695 and 830 nm; 2 mW for each wavelength) and sampled at a rate of 10 Hz. The obtained data were analyzed using the modified Beer–Lambert law for a highly scattering medium (Cope et al., 1988). Changes in oxygenated hemoglobin (oxy-Hb) and deoxygenated hemoglobin (deoxy-Hb) signals were calculated in units of millimolar–millimeter (Maki et al., 1995).

#### fNIRS Data Analyses

For fNIRS data analyses, first, three participants who had poor data quality (mostly due to insufficient probe contact), and two participants with data corruption were excluded, as mentioned above. Consequently, 53 participants were used for the fNIRS analyses. Then, in order to properly detect functional activation, all the collected individual fNIRS data were preprocessed so as to remove temporally colored noise (Uga et al., 2014). Individual time series data for the oxy-Hb and deoxy-Hb signals of each channel were preprocessed using the Wavelet-Minimum Description Length detrending algorithm to remove global trends due to physiological (cardiac, respiratory, and vasomotor-related) fluctuations and other experimental errors (Jang et al., 2009) and then using temporal smoothing with convolution of the canonical hemodynamic response function (HRF) to the individual time series data (Friston et al., 2000).

Next, first-level analyses of fNIRS data collected using an event-related design were performed by regressing the fNIRS signal on a general linear model (GLM) constructed by convolving the expected HRF with a boxcar function representing the temporal structure of the experimental condition. Standard neuroimaging analysis tools, such as statistical parametric mapping (SPM^2^), have adopted HRF based on the convolution of the boxcar function and the sum of two gamma functions as the canonical HRF. When HRF is convolved to a boxcar function representing the temporal structure of an experimental design, a temporal delay (typically 6 s) is incorporated into the GLM analyses since there is a time lag between a neuronal event and the subsequent hemodynamic response, and it is accepted practice to use default temporal parameters such as peak latencies of the gamma functions, which was proposed by Boynton et al. (1996), to describe the observed blood-oxygen-level-dependent (BOLD) signal in response to neural activity. However, hemoglobin signal may differ depending on participant age, although the canonical HRF parameters used in functional magnetic resonance imaging (fMRI) analyses with GLM are well suited to hemodynamics in adults, or they may vary depending on the brain region, and/or the experimental condition used. Since it was unclear whether it would be appropriate to apply the same model to the present study, we attempted to adjust the temporal parameters of a GLM for the fNIRS signals obtained in the present study. Thus, we employed a GLM-based method utilizing an adaptive HRF by varying temporal delay parameters. The optimal temporal parameters were investigated to identify the best-fit time series data during passive sentence listening.

Specifically, individual time series data for the oxy-Hb and deoxy-Hb signals of each channel were analyzed using the GLM with regression to the following HRF, *h*(*τ_p_*,*t*), proposed by Friston et al. (1998).

$$h(\tau_{p},t)=\frac{t^{\tau_{p}}e^{-t}}{(\tau_{p})!}-\frac{t^{\tau_{p}+\tau_{d}}e^{-t}}{A(\tau_{p}+\tau_{d})!}$$

where *t* stands for a point in the time series. The double-gamma function is expressed with two components: the first term is the positive gamma function indicating hemodynamic response and the second term indicates a small undershoot of the hemodynamic response on recovery. The parameter *τ_p_* stands for the first peak latency, and *τ_p_* + *τ_d_* is the second peak (small undershoot) latency, which means that *τ_d_* is the second peak latency from the time point of the first peak latency *τ_p_*. *A* is the amplitude ratio between the first and second peaks. Basically, *τ_p_* is set to 6 s in most fMRI studies since the default setting of the widely used SPM is as follows: (*τ_p_*, *τ_d_*, *A*) = (6, 10, 6). We modified the canonical HRF by adjusting the two gamma functions. The first peak latency, *τ_p_*, was set as a variable by systematically changing it from 3 to 20 s to yield the optimal HRF. In order to avoid complication, the second peak delay *τ_d_* and amplitude ratio *A* were set to the typical default values. Thus, the HRF parameters used in the present study is as follows: and (*τ_p_*, *τ_d_*, *A*) = (3–20, 10, 6).

**FIGURE 1:**
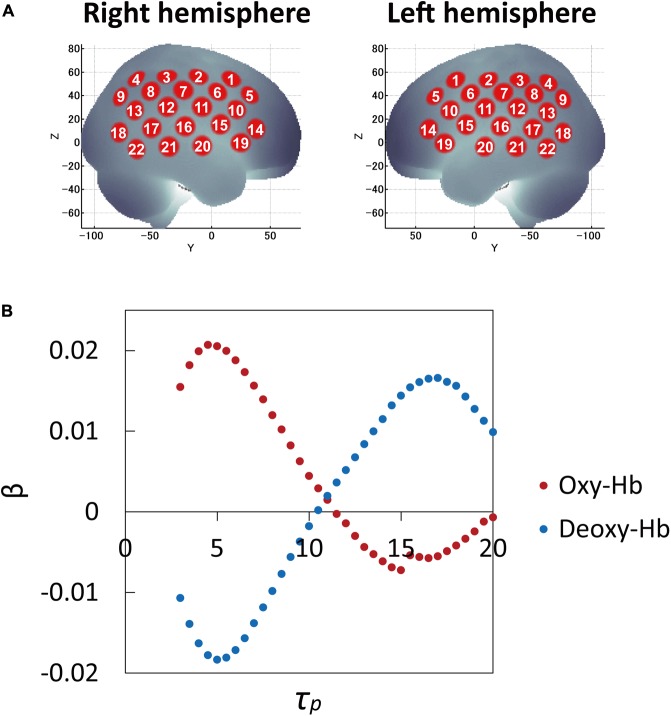
Cortical projection points of fNIRS measurements and average β-values (response amplitudes) of the oxy-Hb and deoxy-Hb signals over 53 participants and three conditions as a function of peak latency *τ_p_*. **(A)** Cortical projection points of fNIRS measurements (location of 22 channels on each hemisphere) are mapped onto the MNI standard brain coordinate system using spatial registration. **(B)** To examine the effects of *τ_p_*, the average β-values over 53 participants and three conditions were calculated and compared for all *τ_p_* ranges for 44 channels. Channel 16 in both the left and right hemispheres in the vicinity of auditory cortex were included among the channels within the top 10% β-values of 22 channels in each hemisphere. Therefore, *τ_p_* was determined by averaging the *τ_p_* values at the highest β-values of channel 16 in both hemispheres: optimal *τ_p_* was 5 s for both oxy-Hb and deoxy-Hb signals.

The β-values (response amplitudes) of the oxy-Hb and deoxy-Hb signals were calculated using a least-squares-model fitting procedure maximizing model-to-data fitting (Bullmore et al., 1996a,b). To examine the effects of *τ_p_*, the average β-values over 53 participants were calculated for all *τ_p_* ranges for 44 channels and three conditions. While the average β-values as a function of peak latency *τ_p_* for three conditions (correct, incorrect, and filler sentences) showed similar curve patterns, those between channels (brain regions) varied. Thus the average β-values over three conditions for 44 channels were examined and compared. Channel 16 in both the left and right hemispheres, in the vicinity of the auditory cortex, were included among the channels with the highest 10% β-values of the 22 channels in each hemisphere. Therefore, the *τ_p_* was determined by averaging the *τ_p_* values at the highest β-values of channel 16 for both hemispheres. **Figure 1** shows average β-values for oxy-Hb and deoxy-Hb signals over 53 participants and three conditions as a function of peak latency *τ_p_*, which was used to determine *τ_p_*. Thus the optimal *τ_p_* was found to be 5 s for both oxy-Hb and deoxy-Hb signals.

The obtained β-values were subjected to second-level group analyses. The group analyses focused on changes in oxy-Hb because of a higher signal-to-noise ratio and a stronger correlation with BOLD signals measured by fMRI (Strangman et al., 2002), a higher sensitivity to changes in cerebral blood flow than are observed for deoxy-Hb and total-Hb signals (Hoshi et al., 2001; Hoshi, 2003), and a higher retest reliability (Plichta et al., 2006).

Statistical analyses were carried out using the SPSS statistical package (SPSS, Chicago, IL, United States). For fNIRS data, we first conducted a statistical analysis to examine significantly activated channels (β-values) using one-sample *t*-tests for all the participants. Then, as significant differences in the behavioral performance were identified between boys and girls, we conducted correlation analyses between behavioral test and cortical activation separately for both sexes. Correlation analyses were performed for correct- and incorrect-sentence conditions, respectively. We used Bonferroni correction by applying the Dubey/Armitage-Parmar (D/AP) alpha boundary (Sankoh et al., 1997) to take into account the spatial correlation of 44 measurement channels. The D/AP procedure has been applied in previous fNIRS studies (Plichta et al., 2006; Sassaroli et al., 2008; Schecklmann et al., 2014; for a review, see Tak and Ye, 2014). The mean correlation coefficient between the channels in all conditions for boys and girls was 0.385, and the resultant adjusted alpha level determined with the D/AP procedure was 0.005. Thus, we set the statistical threshold of fNIRS analysis at 0.005. In order to consider the spatial extent of cortical activation, we defined regions of interest (ROIs) that consisted of single or multiple core channels which fulfilled the determined threshold (0.005) and of adjacent channels that satisfied a secondary threshold of *P* < 0.05.

Regarding anatomical location of measurement channels, we used a probabilistic registration method (Singh et al., 2005) to register average fNIRS data obtained from all participants to the Montreal Neurological Institute (MNI) standard brain space. We referred to the following anatomical atlases: AAL, Brodmann’s atlas, and LPBA40 (Lancaster et al., 2000; Tzourio-Mazoyer et al., 2002; Shattuck et al., 2008).

## Results

### Behavioral Results—All Participants

Because the maximum scores differed across tests (KET_L 25, KET_RW 60, Grammar_L 48, and Grammar_R 15), raw scores were converted into percentages and are presented as such for the sake of uniformity. Mean accuracy (%) and standard deviations (mean ± SD) of the four language tests are given as descriptive statistics: KET_L 43.25 ± 22.16, KET_RW 42.89 ± 19.02, Grammar_L 54.44 ± 13.31, and Grammar_R 75.60 ± 21.04. Correlation analyses showed significant correlations between participants’ scores on all four language tests. Specifically, Grammar_L score was significantly correlated with scores of Grammar_R (correlation coefficient: *r* = 0.451, *P* < 0.001), KET_L (*r* = 0.605, *P* < 0.001), and KET_RW (*r* = 0.551, *P* < 0.001). This suggests that the Grammar_L score, which used the same correct and incorrect sentences as presented auditorily during brain activity measurements, predicts overall L2 (English) performance.

### Behavioral Performance—Examining Sex Differences

In our previous study of elementary school children aged 6–10 years, we found sex differences in L2 word processing (Sugiura et al., 2015). Although the ages (elementary school children vs. junior high school students) and the experimental conditions (word vs. sentence) examined in the previous and present studies are different, we focused on the sex effect in the present study.

**FIGURE 2:**
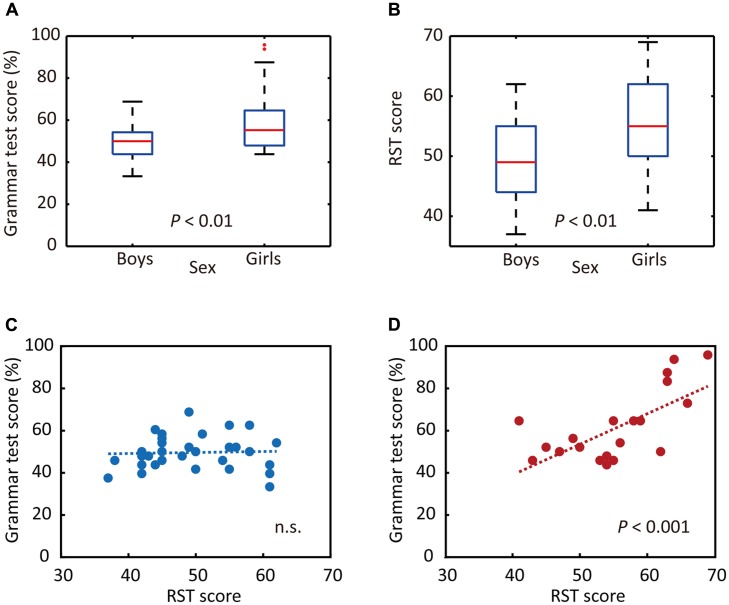
Sex differences in behavioral responses. **(A)** Sex differences in grammar listening test (Grammar_L) scores. **(B)** Sex differences in RST scores. **(C)** Relationship between RST and Grammar_L scores for boys. **(D)** Relationship between RST and Grammar_L scores for girls. While there was no significant relationship between RST and Grammar_L scores for boys **(C)**, there was a significant positive relationship between them for girls **(D)**.

To that end, we first examined whether junior high school boys and girls exhibited different performance on the auditory L2 tests. The results of statistical analyses indicated that girls obtained significantly higher scores for Grammar_L (*P* = 0.004, *d* = 0.98, **Figure 2A**) as well as for KET_L (*P* = 0.02, *d* = 0.76). We also investigated whether the RST scores differed between sexes. The mean RST score of all participants was 51.89 ± 7.85 (mean ± SD, where the maximum score is 70). The statistical analysis indicated that girls outperformed boys on the RST (*P* = 0.005, *d* = 0.82, **Figure 2B**). In sum, significant sex differences were identified for performance on the Grammar_L (assessing grammatical knowledge using the same sentences as presented for brain measurements), KET_L (assessing comprehensive listening ability), and RST (as an index of WM capacity) tests, on all of which the girls obtained higher scores than the boys.

Then, we examined whether the RST scores correlated with those of Grammar_L. Separate analyses were done for boys and girls, as prior analyses indicated significant sex differences for these test scores. As shown in **Figures 2C,D**, the results of single regression analyses indicated that there were significantly positive correlations between scores for RST and Grammar_L (*r* = 0.672, adjusted coefficient of determination: adjusted *r*^2^ = 0.424, *P* < 0.001, **Figure 2D**) for girls, whereas no significant correlations appeared for boys [*r* = 0.042, adjusted *r*^2^ = -0.033, *P* = 0.822 (n.s.), **Figure 2C**]. These results indicate that girls with a higher WM capacity attained significantly higher L2 test scores than did boys and girls with a lower WM capacity.

### ERP Results—Electrophysiological Responses at the Violation Point in L2 Sentences

**FIGURE 3:**
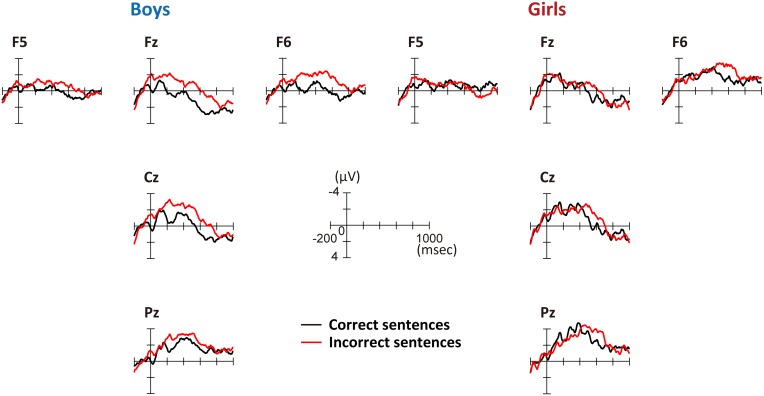
Grand-average ERPs at the onset of the second phrase. Five exploring electrodes were placed based on the international 10–20 system. Negative voltage is plotted up. Red lines denote the average amplitude for incorrect sentences and black lines denote that for correct sentences. **Left**: grand-average ERPs for boys; **right**: grand-average ERPs for girls.

**FIGURE 4:**
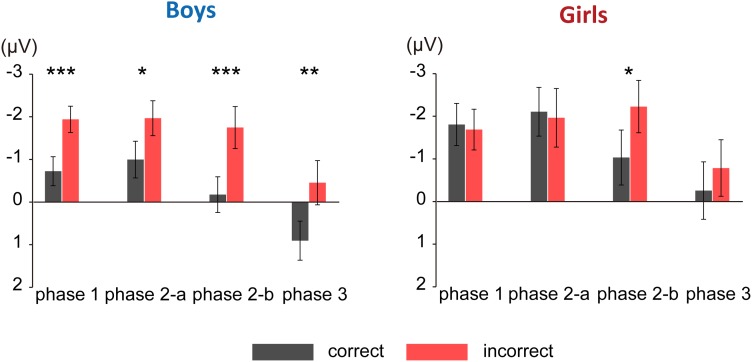
Mean amplitude of each phase. The mean amplitudes of the five exploring electrodes are plotted for each phase. **Left**: mean amplitude for boys; **right**: mean amplitude for girls. Black bars denote amplitude for correct sentences. Red bars denote amplitude for incorrect sentences. Asterisks represent statistical significance in *post hoc* comparisons (^∗∗∗^*P* < 0.005, ^∗∗^*P* < 0.01, ^∗^*P* < 0.05). Error bars indicate standard error.

**Figure 3** shows the grand average ERPs for correct and incorrect sentences, comparing boys and girls. There is a negative shift in incorrect sentences with an early timing in boys. In **Figure 4**, the mean amplitudes of the five exploring electrodes in each phase are plotted for boys and girls, respectively. We first performed ANOVAs for each phase (sex × grammaticality × electrodes). In phase 1, there was an interaction between sex and grammaticality [*F*(1,51) = 5.238, *P* = 0.026, $\eta_{p}^{2}$ = 0.09]. The mean amplitude of incorrect sentences was more negative than that of correct sentences only in boys (*P* = 0.002, *d* = 0.67). Phase 2-a did not show any grammaticality effect, but in phase 2-b, a main effect of grammaticality was observed [*F*(1,51) = 13.467, *P* = 0.001, $\eta_{p}^{2}$ = 0.21]. A main effect of grammaticality was also observed in phase 3 [*F*(1,51) = 7.278, *P* = 0.009, $\eta_{p}^{2}$ = 0.16]. We further applied ANOVAs (grammaticality × electrodes) to each phase for boys and girls, respectively. For boys, main effect was significant for grammaticality in every phase [phase 1: *F*(1,30) = 9.964, *P* = 0.004, $\eta_{p}^{2}$ = 0.25, phase 2-a: *F*(1,30) = 4.612, *P* = 0.04, $\eta_{p}^{2}$ = 0.13, phase 2-b: *F*(1,30) = 9.556, *P* = 0.004, $\eta_{p}^{2}$ = 0.24, and phase 3: *F*(1,30) = 8.193, *P* = 0.008, $\eta_{p}^{2}$ = 0.22]. On the other hand, girls showed a main effect of grammaticality only in phase 2-b [*F*(1,21) = 4.982, *P* = 0.037, $\eta_{p}^{2}$ = 0.19]. These ERP results suggest that boys were sensitive to the syntactic violations in L2.

### fNIRS Results—Hemodynamic Responses during L2 Sentence Processing

**FIGURE 5:**
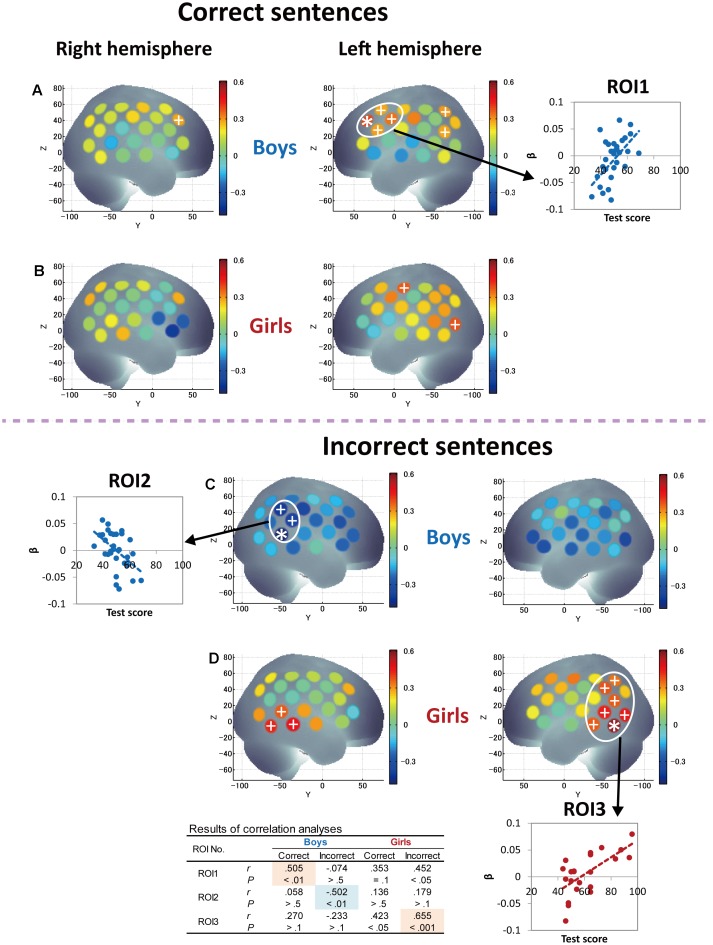
Correlations between grammar listening test scores and cortical activation during correct-sentence processing [boys **(A)** and girls **(B)**] and incorrect-sentence processing [boys **(C)** and girls **(D)**]. Colored bars represent Pearson’s correlation coefficient. Asterisks depict channels that showed a significant correlation between test score and cortical activation after Bonferroni correction using the Dubey/Armitage-Parmar (D/AP) alpha boundary to take into account the spatial correlation of 44 measurement channels. We set the statistical threshold of fNIRS analysis at 0.005. In order to consider the spatial extent of cortical activation, we defined ROIs that consisted of single or multiple core channels which fulfilled the determined threshold (0.005) and adjacent channels that satisfied a secondary threshold of *P* < 0.05, which are depicted with plus signs. The average activation of the nearest-neighboring significant channels satisfying the above threshold was calculated for each ROI, and graphs showing the correlations between test score and cortical activation are displayed. The table at the bottom shows the statistical results of correlation analyses (Pearson’s correlation coefficients, *r* and *P*-values) for each ROI so that the trends of similarities and/or differences in the relationships between Grammar_L scores and cortical activation can be compared between sexes, as well as between correct- and incorrect-sentence conditions.

We examine correlations between L2 proficiency and cortical responses using separate analyses for boys and girls. First, the correlations between Grammar_L score and cortical activations during correct- and incorrect-sentence processing were examined. The results are shown in **Figure 5**, in which magnitudes of Pearson’s correlation coefficients are rendered on a standard brain surface. As for correct-sentence processing (**Figures 5A,B**), both boys and girls had an increased degree of activation in the frontal region, including Broca’s area, and posterior language regions as proficiency increased. However, while activation in boys predominantly increased in anterior cortical regions with proficiency (**Figure 5A**), activation in girls increased relatively in posterior cortical regions, including the superior and middle temporal gyri (STG and MTG; Wernicke’s area), angular gyrus (AG), and supramarginal gyrus (SMG) (**Figure 5B**), as proficiency increased. Note that increased activation in the left hemisphere relative to the right hemisphere was observed with proficiency for both sexes, suggesting that left-lateralized activation with L2 development is common to both sexes.

Next, correlations between Grammar_L score and cortical activations during incorrect-sentence processing were examined and compared with those during correct-sentence processing. The results are described in **Figures 5C,D**. Intriguingly, boys and girls had totally different changes in response to incorrect sentences with proficiency. Specifically, girls exhibited a positive correlation between the test score and cortical activation in the broad region, and significant correlations were observed mainly in the posterior language regions, including STG, MTG, AG, and SMG (**Figure 5D**), which was the same pattern observed for correct-sentence processing. In contrast, boys exhibited a negative correlation between test score and cortical activation for all the cortical regions examined (**Figure 5C**).

**FIGURE 6:**
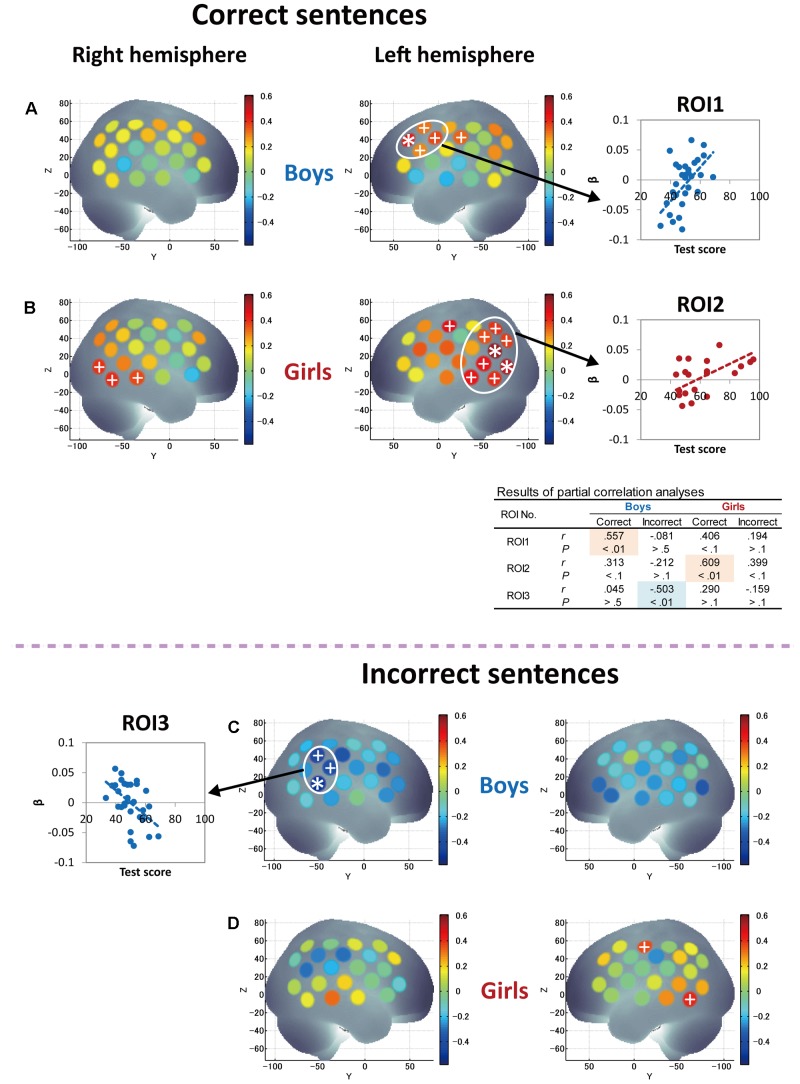
Results of partial correlation analyses between grammar listening test score and cortical activation during correct-sentence processing [boys **(A)** and girls **(B)**] and incorrect-sentence processing [boys **(C)** and girls **(D)**] with RST score (WM capacity) as a control variable. Colored bars represent partial correlation coefficient. Asterisks and plus signs are the same as those in **Figure 5**. In the table to the middle right side, statistical results of partial correlation analyses (partial correlation coefficients, *r* and *P*-values) are shown for each ROI so that the trends of similarities and/or differences in the relationships between Grammar_L scores and cortical activation can be compared between sexes, as well as between correct- and incorrect-sentence conditions.

Finally, since significant correlations between RST and L2 test scores were identified only in girls (**Figures 2C,D**), we further attempted to derive the pure characteristics of language processing and compare those characteristics between boys and girls by considering the effects of WM capacity. Thus, we conducted partial correlation analyses using RST score (the index of WM capacity) as a control variable to derive more language-specific cortical activation from the fNIRS data. The results of partial correlation analyses between Grammar_L score and cortical activation during sentence processing are shown in **Figure 6**. With regard to correct-sentence processing (**Figures 6A,B**), the overall results were the same as those of correlation analyses described in **Figures 5A,B**, indicating more left-lateralized activation with proficiency in both sexes. However, the results of the partial correlation analyses revealed further significant differences between boys and girls: as proficiency increased, while boys had significantly increased activation in the anterior compared to the posterior cortical region (**Figure 6A**), girls had significantly increased activation in a broad posterior cortical region (**Figure 6B**). Regarding incorrect-sentence processing (**Figures 6C,D**), the statistical results for boys (**Figure 6C**) were almost identical to those of the correlation analyses shown in **Figure 5C**, and for girls, there were no significant channels after adjustment for multiplicity (**Figure 6D**).

## Discussion

Individual differences, such as sex, individual abilities, state, traits, etc., seem to have greater effects on L2 compared to L1, while developmental sequences for L2 may be similar to those for L1 (Dulay and Burt, 1974; Hatch and Wagner-Gough, 1976) and analogous brain regions are recruited for L1 and L2 (e.g., Rüschemeyer et al., 2006; Suh et al., 2007; for reviews, see Abutalebi, 2008; Kotz, 2009). Significant sex differences in cortical activation were identified for L2 (but not for L1) word processing in elementary school children, and these differences emerged as L2 proficiency increased (Sugiura et al., 2015). In the present study, we applied simultaneous fNIRS–ERP measurements to examine neural responses during L2 syntactic processing at the sentence level as a function of L2 proficiency in young adolescents by considering sex and WM capacity. We hypothesized that adolescent L2 learners have a high level of activation in the left temporal and frontal language areas (Wernicke’s area and Broca’s area) during sentence-level processing as L2 proficiency increases, and that sex differences may appear in a grammaticality effect in ERP and cortical activation. Our findings support this hypothesis; neural responses during syntactic processing of L2 sentences are modulated by L2 proficiency and WM capacity, which show marked sex differences.

### L2 Test and RST Scores

First of all, the behavioral results revealed that girls significantly outperformed boys in the L2 tests as well as in the RST (**Figures 2A,B**). Second, while L2 test scores significantly correlated with RST scores in girls, no correlations were found in boys (**Figures 2C,D**), suggesting that girls with higher WM capacity are more likely than boys and girls with lower WM capacity to rely on WM function to process L2 auditory sentences. This intriguing sex difference may imply that boys and girls have different strategies for L2 sentence processing.

### ERP Findings

The ERP results revealed significant differences in amplitude between correct- and incorrect-sentence processing in every phase [phase 1 (100–300 ms), phase 2-a (300–450 ms), phase 2-b (450–600 ms), and phase 3 (600–800 ms)] in boys, whereas significant difference was observed only in phase 2-b in girls (**Figure 4**). Importantly, significant differences in the amplitude between correct- and incorrect-sentence processing in phases 1 and 2-a were observed in boys, but not in girls. Given previous evidence that these time windows are indices of syntactic processing, it would appear that boys, relative to girls, were primarily responsive to rule-based syntactic processing. More specifically, the ERP component observed during phase 1 in boys is likely a consequence of automatic, initial syntactic phrase structure processing (ELAN), which is consistent with the current fNIRS results showing that boys have significantly increased activation in the left frontal operculum (BA44) with proficiency. The amplitude difference observed in the subsequent time window (phase 2-a) also supports the notion of an “innate” syntactic processing in boys because the LAN component reflects structural processing, including verb argument structure, which is related to thematic role assignment (Friederici, 2002). This means that the boys were sensitive to the structure of simple sentences in English and might have strongly expected a verb to follow an initial animate noun which could be the subject of the sentence.

Previous ERP studies using phrase structure violation sentences often observed a biphasic ELAN (LAN)—P600 pattern (e.g., Hahne and Friederici, 1999, 2002; Rossi et al., 2006). It is, however, interesting to note that, in the present study, neither group showed any positive effect, including during the P600 time window. The P600 component, which involves controlled processing unlike ELAN, reflects syntactic reanalysis or repair processes. Alternatively, boys showed a sustained negativity until phase 3. It is difficult to clearly distinguish the end of ELAN/LAN components within the present sustained negativity, but this negative effect was mainly observed in fronto-central electrodes, suggesting that the present anterior negativity indicates an effect continuation of ELAN/LAN components and a failure to achieve the explicit controlled syntactic processing during, for example, the P600 component. Interestingly, similar sustained negativity was also reported in an ERP study of children’s language development (Hahne et al., 2004), which revealed a similar aspect of the neural basis of L1/L2 syntactic development.

As for girls, there were no significant differences in response to correct and incorrect sentences in phases 1, 2-a, and 3, suggesting that girls were less likely to fully engage in syntactic processing when they heard incorrect sentences. Instead, the ERP result suggests that the girls focus on semantic processing. The amplitude differences that were identified only in phase 2-b in girls, which were observed in anterior to posterior electrodes, are considered to be an N400 component. Some L2 ERP studies have shown that L2 learners elicit an N400, but not P600, component in syntactically anomalous sentences (e.g., Hahne and Friederici, 2001; Weber and Lavric, 2008). Further, it should be noted that although a standard N400 effect is often seen in the 300–550 ms latency range, it has been identified with reduced amplitudes and delayed latencies in L2 learners (Hahne, 2001; Mueller, 2005). This ERP result showing that girls tend to rely on semantic attributes relative to boys is also in line with our fNIRS results.

### fNIRS Findings

The present fNIRS data identified sex commonalities and differences in response to passive L2 sentence listening. Note that a significant increase in activation in the left hemisphere relative to the right hemisphere was observed with proficiency during correct-sentence processing for both sexes (**Figures 5A,B**, **6A,B**), suggesting that left-lateralized activation with L2 development is common to both sexes. Also, both sexes, but especially boys, exhibited cortical activation in the prefrontal cortex encompassing Broca’s area during the passive sentence listening. Given these results, the prefrontal region can be identified as being involved not only in language production, but also in comprehension in the early stage of L2 learning, and the current results demonstrate that fundamental aspects of L2 comprehension are processed by a shared neural network that also supports L1 processing. Thus, the present study clarified common aspects of language processing in L2 irrespective of sex. However, at the same time, sex differences manifested: as proficiency increased, boys had significantly increased activation in the prefrontal region, while girls predominantly had increased activation in the posterior language region, including the STG/MTG (Wernicke’s area), AG, and SMG, during correct-sentence processing. This trend was more significant especially after removing the effect of WM function (**Figures 6A,B**).

A number of previous lesion studies suggested that the left anterior brain regions are involved in syntactic processing, whereas the posterior brain regions, especially left temporal regions, are thought to process lexical semantics (Caplan, 1992; Goodglass, 1993; Grodzinsky, 2000); thus, two functionally distinctive regions, syntactic knowledge (rule-based grammatical knowledge) and lexical knowledge (word forms and meanings), had long been postulated. However, modern functional neuroimaging techniques have allowed remarkable advances in our understanding of brain–language relationships. A large-scale meta-analysis utilizing the results from 129 scientific reports, defined the composition of phonological, semantic, and sentence processing networks in the frontal, temporal, and inferior parietal regions of the left cerebral hemisphere, and updated the view of brain–language relationships (Vigneau et al., 2006). The results revealed distinct (although partially overlapping) networks for phonology, semantics, and sentence processing, and, importantly, all three language processes are supported by fronto-temporal networks with distinct, but partially overlapping, areas.

According to their results, the posterior temporal and parietal regions are related to all three language processes, with phonological clusters located in the STG and SMG, semantic clusters located in the STG, MTG, and AG, and sentence clusters located in the posterior portion of the STG as well as in the posterior part of the MTG. This proposal is consistent with previous work: the AG and SMG in the parietal region are known to be involved in semantic and phonological processing, respectively, and the SMG has also been shown to play a role in the retrieval and association of semantic knowledge (Damasio, 1990; Vandenberghe et al., 1996; Murtha et al., 1999; Wiggs et al., 1999). In contrast, the frontal region also supports all three language processes with phonological clusters located in a more caudal position in the frontal lobe, semantic clusters located in the anterior part of the inferior frontal gyrus (IFG), and sentence clusters located in the posterior part of the middle frontal gyrus and in the dorsal and upper part of the pars opercularis.

In another study, Friederici et al. (2003) reported brain areas for the processing of sentence-level semantic and syntactic information using an event-related fMRI paradigm. They found that processing of semantic violations at the sentence level relied primarily on the superior temporal region bilaterally, whereas processing of syntactic violations in sentences specifically involves the left posterior frontal operculum adjacent to Broca’s area. Consistent results have been reported for semantic processing (Caplan et al., 1998; Kuperberg et al., 2000; Ni et al., 2000) and for syntactic processing (Just et al., 1996; Stromswold et al., 1996; Caplan et al., 1998, 1999; Dapretto and Bookheimer, 1999; Embick et al., 2000; Friederici et al., 2000). They also mentioned that the left frontal operculum in the IFG (BA44) is responsible for on-line syntactic phrase structure building processes during auditory comprehension. In our study, boys had significantly increased activation with proficiency mainly in the left frontal operculum. Given the previous reports, boys are likely to engage in on-line syntactic phrase structure building processing during L2 sentence listening. With regard to the posterior STG, Friederici et al. (2003) identified that both sentence-level semantically and syntactically anomalous conditions generated greatly increased activation in comparison to correct sentences. Our results in girls are in line with their results in that girls had increased activation in the posterior STG with proficiency in sentence processing irrespective of sentence type (**Figures 5B,D**), and, importantly, their activation was significantly more increased in the incorrect-sentence condition (**Figure 5D**) than in the correct-sentence condition (**Figure 5B**). Since girls had significantly increased activation not only in the STG, but also in the MTG, AG, and SMG, it is highly possible that they process sentences through a consolidation of phonological, semantic, and sentential information.

### Sex Differences Observed in fNIRS and ERP

With regard to incorrect-sentence processing, interesting sex differences were revealed in the fNIRS data. Girls had significantly increased activation with proficiency in the left posterior temporal and parietal regions in the incorrect-sentence condition (**Figure 5D**), similar to but more obvious than increased activation in the correct-sentence condition (**Figure 5B**), with accompanying increased activation in the right posterior temporal region (**Figure 5D**). Conversely, boys had decreased activation with proficiency in almost all brain regions examined, especially in the right homolog of Wernicke’s area (**Figure 5C**). The observed diametrical responses during passive incorrect-sentence listening were beyond our expectations, but are very interesting. As proficiency increases, boys may tend to goof off as they hear an incorrect sentence, and this response is completely different from that in the case of correct-sentence processing. Importantly, this distinction between the two conditions implies that boys are increasingly able to distinguish between correct and incorrect sentences with proficiency, irrespective of their poor performance in the Grammar_L compared to the girls. Intriguingly, the differences in the response to the correct- and incorrect-sentence conditions in boys (**Figure 5A** vs. **Figure 5C**) were more remarkable than those in girls (**Figure 5B** vs. **Figure 5D**). More importantly, ERP data demonstrated that boys had a faster response than girls in distinguishing between correct and incorrect sentences, showing ELAN/LAN reflecting syntactic processing. Given the evidence available, we can postulate that boys may preferentially engage in rule-based syntactic processing while listening to a mixture of correct and incorrect sentences, and that their brains may respond to the grammatical differences in an implicit manner during sentence listening. Thus, at the beginning of L2 learning as a mandatory academic subject in junior high school, boys are behaviorally less likely to explicitly distinguish between grammatically (syntactically) correct and incorrect sentences compared to girls, but they may develop syntactic competence before being aware of it.

Previous studies have indicated that rule-based syntactic processing depends on procedural memory supported by a basal ganglia-frontal lobe system, while lexical memory depends on declarative memory supported by a temporal/temporo-parietal system (Ullman et al., 1997; Ullman, 2001). The procedural memory system is known to support a variety of well-established motor, perceptual and cognitive skills, and through this system, we implicitly acquire, store, and use knowledge. Thus, it is plausible that rule-based syntactic processing is also supported by this system. Furthermore, as mentioned in the paper by Lum et al. (2012), who examined multiple memory systems and their interactions in relation to language functions, learning in procedural memory is slower than in declarative memory: it proceeds gradually, as stimuli are repeated and skills practiced. It is quite conceivable that boys are slower at learning syntactic metaknowledge than girls if they rely on procedural memory to acquire rule-based grammatical knowledge, and that their brains may implicitly respond to differences between correct and incorrect sentences at the beginning of learning. This view is further supported by the ERP data. Previous ERP work suggested that early ERP components are best explained by a model with feedforward connections only and that backward connections become essential only after 220 ms (Garrido et al., 2007) because there is not enough time for return activity to pass from higher-level to low-level brain areas (e.g., ELAN). Given that significant differences in ERP responses were observed between the correct- and incorrect-sentence conditions during phase 1 before 220 ms in boys (**Figure 3**, left side) combined with all the previous information available, it seems that boys are likely to implicitly detect differences in phrase structure between correct and incorrect sentences, or syntactic phrase structure violations, in an automatic manner even though their implicit awareness is not reflected in their behavioral performance (L2 tests scores).

In contrast to boys, girls with higher L2 proficiency seem to be earnest in comprehending both incorrect and correct sentences. They imposed an even greater activation load with proficiency in the incorrect-sentence condition (**Figure 5D**) than in the correct-sentence condition (**Figure 5B**), with increased activation in the bilateral temporal and the left parietal regions. Note that not only the prefrontal region, but also the posterior language region have been reported to exhibit increased activation with WM loads (Vigneau et al., 2006). A previous study revealed a dissociation of activation in two cortical regions in the WM network: a major role of the anterior region is monitoring information, whereas a crucial role of the posterior region is manipulating information in WM (Champod and Petrides, 2010). The results of our partial correlation analyses provided valuable additional information. After deriving more language-specific cortical activation by removing the WM function effect, the increased activation in the posterior STG with proficiency observed in incorrect-sentence processing in girls (**Figure 5D**) was less prominent (**Figure 6D**). This suggests that the significant increase in posterior STG activation for incorrect-sentence processing (**Figure 5D**) was a consequence of increased WM load. Incorrect-sentence processing may be more difficult than correct-sentence processing, when sentences are presented auditorily, leading to greater activation for incorrect- than for correct-sentence conditions. Given previous findings, girls are very likely to process sentences by drawing on all available functions: phonological, semantic, sentential processing, and the respective WM subsystems, and this is probably why their RST and L2 test scores were closely correlated (**Figure 2D**). Since girls significantly outperformed boys in the L2 grammar test, and they tend to preferentially focus on sentence comprehension by considering semantic aspects, they seem to explicitly distinguish between correct and incorrect sentences.

Sex differences in language performance are debated in both behavioral and neuroscience studies, which often provide results indicating female superiority; however, it is a controversial topic. Most previous studies regarding sex differences have found such differences, but do not or cannot provide a comprehensive understanding of the details of and mechanisms underlying the pertinent brain functions. The present study regarding young adolescents provides a comprehensive understanding of this issue. By using fNIRS and ERP simultaneously, the present study has produced consistent results: ERP results show higher sensitivity to syntactic violations among boys and higher sensitivity to semantics among girls, results which are validated with fNIRS findings of activations in the IFG BA44 and the STG/MTG/SMG/AG, respectively. Girls overall had explicitly better scores than boys on the L2 grammar test, which is in line with previous studies showing female superiority. However, if we had looked only at their behavior, we would not have been able to elucidate the details of the underlying strategies and brain activity of both sexes, such as the point that boys are implicitly aware of syntactic structure irrespective of their inferior performance compared to girls on the L2 grammar test.

Combining both behavioral and neuroimaging data, we demonstrated sex differences in L2 sentence processing; this finding is not a judgment about which is better or worse, but rather an opportunity to deepen our understanding of the individual differences in strategies for language learning. It is quite understandable that on average, there are differences in the strategies preferred by boys and girls: the strategies they employ are respectively more likely to allow them success in L2 learning. Boys seem to engage early on during L2 learning in implicit, rule-based syntactic processing; conversely, girls seem to rely on a myriad of cognitive functions during L2 processing, allowing better overall explicit language performance despite less automaticity in detecting syntactic errors. While it may be reasonable to draw on all available knowledge and functions (phonological, semantic, and syntactic) simultaneously during sentence processing for better understanding if one has sufficient WM capacity, it may make more sense for some individuals to focus only on singular points (e.g., violation points) or rule-based syntactic processing in order to reduce the load imposed on their WM. Alternatively, boys may simply prefer efficient strategies regardless of WM capacity.

The present findings provide insight into the mechanisms behind how junior-high-school aged boys and girls master an L2. They may also contribute to future L2 education by providing a foundation upon which to base thorough and meticulous approaches that will open the way for effective teaching methods that take sex and/or individual differences into consideration in school education, for example, an approach that bolsters intuitive structural processing for girls and the ability to retain the accumulative information in sentences for boys; and these and subsequent findings will allow the development of English learning methods based on cognitive neuroscience evidence.

### Sex Differences in Working Memory and L2 Performance

Lastly, with regard to sex differences in WM capacity and a further possibility of its influence upon L2 performance, it is presumable that catechol-*O*-methyltransferase (COMT) Val^158^Met genotype may be relevant. Previous studies have identified that the *COMT* genotype influences WM function (e.g., Bruder et al., 2005; Tan et al., 2007; Vijayraghavan et al., 2007; Diaz-Asper et al., 2008; Sambataro et al., 2009; Cools and D’Esposito, 2011; Stokes et al., 2011), which is implicated in dopamine functioning. Importantly, behavioral studies have indicated an association between WM performance and the *COMT* polymorphism in children and adolescents in a normal population (Diamond et al., 2004; Wahlstrom et al., 2007; Barnett et al., 2008; Dumontheil et al., 2011). In fact, in a former study, we found significant *COMT* genotype effects on language functions in children (Sugiura et al., 2017), and in the present study we have demonstrated some effects of WM function, reinforcing the idea of an association between the two. While we did not find sex differences in the genotype effects in our former study dealing with children 6–10 years of age, the participants in the present study were adolescents aged 12–15 years. Indeed, Barnett et al. (2007) found a significant genotype effect on executive function and verbal IQ, and subsequent analyses including sex as a factor found significant genotype effects only in boys. Importantly, these effects were significantly greater in pubertal than in prepubertal boys. Furthermore, another study of the *COMT* gene in children (Gaysina et al., 2013) assessed verbal and non-verbal cognition at ages 8–15 years using a longitudinal design. In that study, *COMT* was associated with reading comprehension, verbal ability, and global cognition at age 15 years in pubescent boys, but not at age 8. These findings suggest that the sex difference in WM capacity and a further possibility of its influence upon L2 performance observed in the present study may be due to sex differences in the *COMT* genotype effects, although future studies are needed to confirm this assumption.

### Limitations

One limitation of this study is that we could only employ a passive listening task. In a future study, looking at cortical activation during an active listening task (i.e., by asking the participants to detect incorrect sentences) would have the potential to show cortical regions related not only to syntactic processing but also to memory and attention functions for performing the active task (Vannest et al., 2009) and to reveal how actual performance during a task is linked to activations.

In the present study, we used the RST to measure WM capacity, as we thought that it was more closely related to language performance compared to a non-linguistic WM test. However, in addition to the RST, it may be interesting to use a non-linguistic WM test and compare the results.

Despite having more boys than girls among our participants, there were few boys with a high WM capacity comparable to that of girls in this age range. If there had been some boys with a WM capacity comparable to that of girls in the current study, we may have been able to separate the sex factor from the WM factor more precisely. It would be interesting if one could separate these two factors entirely in a future study by including a large number of boys and girls with high WM capacity. It would also be interesting to examine whether the sex differences observed in this study with later L2 learners can be replicated in boys and girls that are early bilinguals.

## Conclusion

Cerebral development with L2 learning was revealed to be similar to that with L1 in regards to the dynamic shift in cerebral dominance to the left hemisphere in sentence processing, and to the analogous language-related brain regions encompassing a fronto-temporal network that are recruited in sentence processing. While the present study consolidated universal characteristics of human language functions, significant sex differences were also revealed. Both boys and girls are assumed to distinguish between syntactically correct and incorrect sentences, but in different manners. During L2 sentence listening, boys generally relied on the prefrontal region implicated in rule-based syntactic processing, suggesting that they tend to focus on processing grammaticality, or phrase structure, while girls generally depended on a broad posterior language-related region involved in phonology, semantics, and sentence processing, suggesting that girls process sentences by consolidating these multiple aspects. The present study also uncovered intriguing sex differences: as proficiency increased, boys had a reduced engagement load, while girls strove to evaluate or process while making the best use of their full linguistic knowledge and WM during grammatically incorrect-sentence listening. By dissociating the effect of sex and removing the effect of WM capacity, significant sex differences in language-specific sentence processing were clarified. At the same time, interesting differences between sexes were also identified in the manner of distinguishing between syntactically correct and incorrect sentences.

## Author Contributions

LS and MH contributed to the design of the study, preparation of behavioral tests and sentence stimuli, acquisition, analysis, and interpretation of data, and writing the paper. HM-K was involved in collecting and organizing data. MU contributed to fNIRS preprocessing. DT was involved in spatial registration of fNIRS data to MNI space. ID contributed to the preparation of the experiment and fNIRS preprocessing. HH supervised the study. FH contributed to the design of the study, programming the experiments, interpretation of data, and revision of the article. All authors approved the final manuscript.

## Conflict of Interest Statement

The authors declare that the research was conducted in the absence of any commercial or financial relationships that could be construed as a potential conflict of interest.

## Footnotes

1 http://surfer.nmr.mgh.harvard.edu/optseq/

2 www.fil.ion.ucl.ac.uk/spm/

## References

[B1] Abutalebi J. (2008). Neural aspects of second language representation and language control. *Acta Psychol.* 128 466–478. 10.1016/j.actpsy.2008.03.014 18479667

[B2] Barnett J. H. Heron J. Ring S. M. Golding J. Goldman D. Xu K. (2007). Gender-specific effects of the catechol-*O*-methyltransferase *Val*^108^/^158^*Met* polymorphism on cognitive function in children. *Am. J. Psychiatry* 164 142–149. 10.1176/ajp.2007.164.1.142 17202556

[B3] Barnett J. H. Scoriels L. Munafo M. R. (2008). Meta-analysis of the cognitive effects of the catechol-*O*-methyltransferase gene Val158Met polymorphism. *Biol. Psychiatry* 64 137–144. 10.1016/j.biopsych.2008.01.005 18339359

[B4] Bauer D. Goldfield B. Reznick S. (2002). Alternative approaches to analyzing individual differences in the rate of early vocabulary development. *Appl. Psycholinguist.* 23 313–335. 10.1017/S0142716402003016

[B5] Baxter L. C. Saykin A. J. Flashman L. A. Johnson S. C. Guerin S. J. Babcock D. R. (2003). Sex differences in semantic language processing: a functional MRI study. *Brain Lang.* 84 264–272. 10.1016/S0093-934X(02)00549-7 12590915

[B6] Boynton G. M. Engel S. A. Glover G. H. Heeger D. J. (1996). Linear systems analysis of functional magnetic resonance imaging in human V1. *J. Neurosci.* 16 4207–4221. 8753882 10.1523/JNEUROSCI.16-13-04207.1996 PMC6579007

[B7] Bruder G. E. Keilp J. G. Xu H. Shikhman M. Schori E. Gorman J. M. (2005). Catechol-*O*-methyltransferase (COMT) genotypes and working memory: associations with differing cognitive operations. *Biol. Psychiatry* 58 901–907. 10.1016/j.biopsych.2005.05.010 16043133

[B8] Bullmore E. T. Brammer M. J. Williams S. C. R. Rabe-Hesketh S. Janot N. David A. (1996a). Statistical methods of estimation and inference for functional MR image analysis. *Magn. Reson. Med.* 35 261–277. 10.1002/mrm.1910350219 8622592

[B9] Bullmore E. T. Rabe-Hesketh S. Morris R. G. Williams S. C. R. Gregory L. Gray J. A. (1996b). Functional magnetic resonance image analysis of a large-scale neurocognitive network. *Neuroimage* 4 16–33. 10.1006/nimg.1996.0026 9345494

[B10] Burman D. D. Bitan T. Booth J. R. (2008). Sex differences in neural processing of language among children. *Neuropsychologia* 46 1349–1362. 10.1016/j.neuropsychologia.2007.12.021 18262207 PMC2478638

[B11] Burman D. D. Minas T. Bolger D. J. Booth J. R. (2013). Age, sex, and verbal abilities affect location of linguistic connectivity in ventral visual pathway. *Brain Lang.* 124 184–193. 10.1016/j.bandl.2012.12.007 23376366 PMC3572208

[B12] Cambria E. White B. (2014). Jumping NLP curves: a review of natural language processing research. *IEEE Comput. Intell. Mag.* 9 48–57. 10.1109/MCI.2014.2307227

[B13] Caplan D. (1992). *Language: Structure, Processing, and Disorders.* Cambridge, MA: MIT Press.

[B14] Caplan D. Alpert N. Waters G. (1998). Effects of syntactic structure and propositional number on patterns of regional blood flow. *J. Cogn. Neurosci.* 10 541–552. 10.1162/089892998562843 9712683

[B15] Caplan D. Alpert N. Waters G. (1999). PET studies of sentence processing with auditory sentence presentation. *Neuroimage* 9 343–351. 10.1006/nimg.1998.0412 10075904

[B16] Champod A. S. Petrides M. (2010). Dissociation within the frontoparietal network in verbal working memory: a parametric functional magnetic resonance imaging study. *J. Neurosci.* 30 3849–3856. 10.1523/JNEUROSCI.0097-10.2010 20220020 PMC6632229

[B17] Chomsky N. (1965). *Aspects of a Theory of Syntax.* Cambridge, MA: MIT Press.

[B18] Clements A. M. Rimrodt S. L. Abel J. R. Blankner J. G. Mostofsky S. H. Pekar J. J. (2006). Sex differences in cerebral laterality of language and visuospatial processing. *Brain Lang.* 98 150–158. 10.1016/j.bandl.2006.04.007 16716389

[B19] Cools R. D’Esposito M. (2011). Inverted U-shaped dopamine action on human working memory and cognitive control. *Biol. Psychiatry* 69 e113–e125. 10.1016/j.biopsych.2011.03.028 21531388 PMC3111448

[B20] Cope M. Delpy D. T. Reynolds E. O. R. Wary S. Wyatt J. van der Zee P. (1988). Methods of quantitating cerebral near infrared spectroscopy data. *Adv. Exp. Med. Biol.* 222 183–189. 10.1007/978-1-4615-9510-6_21 3129910

[B21] Damasio A. R. (1990). Category-related recognition defects as a clue to the neural substrates of knowledge. *Trends Neurosci.* 13 95–98. 10.1016/0166-2236(90)90184-C 1691878

[B22] Daneman M. Carpenter P. A. (1980). Individual differences in working memory and reading. *J. Verb. Learn. Verb. Behav.* 19 450–466. 10.1016/S0022-5371(80)90312-6

[B23] Daneman M. Merikle P. M. (1996). Working memory and language comprehension: a meta-analysis. *Psychon. B Rev.* 3 422–433. 10.3758/BF03214546 24213976

[B24] Dapretto M. Bookheimer S. Y. (1999). Form and content: dissociating syntax and semantics in sentence comprehension. *Neuron* 24 427–432. 10.1016/s0896-6273(00)80855-7 10571235

[B25] Diamond A. Briand L. Fossella J. Gehlbach L. (2004). Genetic and neurochemical modulation of prefrontal cognitive functions in children. *Am. J. Psychiatry* 161 125–132. 10.1176/appi.ajp.161.1.125 14702260

[B26] Diaz-Asper C. M. Goldberg T. E. Kolachana B. S. Straub R. E. Egan M. F. Weinberger D. R. (2008). Genetic variation in catechol-*O*-methyltransferase: effects on working memory in schizophrenic patients, their siblings, and healthy controls. *Biol. Psychiatry* 63 72–79. 10.1016/j.biopsych.2007.03.031 17707347 PMC3708610

[B27] Doran E. W. (1907). A study of vocabularies. *Pedagog. Semin.* 14 401–438. 10.1080/08919402.1907.10532555

[B28] Dulay H. Burt M. (1974). Natural sequences in child second language acquisition. *Lang. Learn.* 24 37–53. 10.1111/j.1467-1770.1974.tb00234.x 26671710

[B29] Dumontheil I. Roggeman C. Ziermans T. Peyrard-Janvid M. Matsson H. Kere J. (2011). Influence of the COMT genotype on working memory and brain activity changes during development. *Biol. Psychiatry* 70 222–229. 10.1016/j.biopsych.2011.02.027 21514925

[B30] Embick D. Marantz A. Miyashita Y. O’Neil W. Sakai K. L. (2000). A syntactic specialization for Broca’s area. *Proc. Natl. Acad. Sci. U.S.A.* 97 6150–6154. 10.1073/pnas.100098897 10811887 PMC18573

[B31] Friederici A. D. (1995). The time course of syntactic activation during language processing: a model based on neuropsychological and neurophysiological data. *Brain Lang.* 50 259–281. 10.1006/brln.1995.1048 7583190

[B32] Friederici A. D. (2002). Towards a neural basis of auditory sentence processing. *Trends Cogn. Sci.* 6 78–84. 10.1016/S1364-6613(00)01839-8 15866191

[B33] Friederici A. D. (2011). The brain basis of language processing: from structure to function. *Physiol. Rev.* 91 1357–1392. 10.1152/physrev.00006.2011 22013214

[B34] Friederici A. D. Gunter T. C. Hahne A. Mauth K. (2004). The relative timing of syntactic and semantic processes in sentence comprehension. *Neuroreport* 15 165–169. 10.1097/00001756-200401190-00032 15106851

[B35] Friederici A. D. Hahne A. Saddy D. (2002). Distinct neurophysiological patterns reflecting aspects of syntactic complexity and syntactic repair. *J. Psycholinguist. Res.* 31 45–63. 10.1023/a:1014376204525 11924839

[B36] Friederici A. D. Meyer M. von Cramon D. Y. (2000). Auditory language comprehension: an event-related fMRI study on the processing of syntactic and lexical information. *Brain Lang.* 74 289–300. 10.1006/brln.2000.2313 10950920

[B37] Friederici A. D. Ruschemeyer S. A. Hahne A. Fiebach C. J. (2003). The role of left inferior frontal and superior temporal cortex in sentence comprehension: localizing syntactic and semantic processes. *Cereb. Cortex* 13 170–177. 10.1093/cercor/13.2.170 12507948

[B38] Friederici A. D. Steinhauer K. Frisch S. (1999). Lexical integration: sequential effects of syntactic and semantic information. *Mem. Cogn.* 27 438–453. 10.3758/BF03211539 10355234

[B39] Friston K. J. Fletcher P. Josephs O. Holmes A. Rugg M. D. Turner R. (1998). Event-related fMRI: characterizing differential responses. *Neuroimage* 7 30–40. 10.1006/nimg.1997.0306 9500830

[B40] Friston K. J. Josephs O. Zarahn E. Holmes A. P. Rouquette S. Poline J. (2000). To smooth or not to smooth? Bias and efficiency in fMRI timeseries analysis. *Neuroimage* 12 196–208. 10.1006/nimg.2000.0609 10913325

[B41] Frost J. A. Binder J. R. Springer J. A. Hammeke T. A. Bellgowan P. S. F. Rao S. M. (1999). Language processing is strongly left lateralized in both sexes: evidence from functional MRI. *Brain* 122 199–208. 10.1093/brain/122.2.199 10071049

[B42] Garrido M. I. Kilner J. M. Kiebel S. J. Friston K. J. (2007). Evoked brain responses are generated by feedback loops. *Proc. Natl. Acad. Sci. U.S.A.* 104 20961–20966. 10.1073/pnas.0706274105 18087046 PMC2409249

[B43] Gaysina D. Xu M. K. Barnett J. H. Croudace T. J. Wong A. Richards M. (2013). The Catechol-*O*-methyltransferase gene (COMT) and cognitive function from childhood through adolescence. *Biol. Psychol.* 92 359–364. 10.1016/j.biopsycho.2012.11.007 23178897 PMC3580283

[B44] Goldstein J. M. Jerram M. Poldrack R. Anagnoson R. Breiter H. C. Makris N. (2005). Sex differences in prefrontal cortical brain activity during fMRI of auditory verbal working memory. *Neuropsychology* 19 509–519. 10.1037/0894-4105.19.4.509 16060826

[B45] Golestani N. Alario F. X. Meriaux S. Le Bihan D. Dehaene S. Pallier C. (2006). Syntax production in bilinguals. *Neuropsychologia* 44 1029–1040. 10.1016/j.neuropsychologia.2005.11.009 16427099

[B46] Goodglass H. (1993). *Understanding Aphasia.* San Diego, CA: Academic Press.

[B47] Grodzinsky Y. (2000). The neurology of syntax: language use without Broca’s area. *Behav. Brain Sci.* 23 1–17. 10.1017/S0140525X00002399 11303337

[B48] Hahne A. (2001). What’s different in second-language processing? Evidence from event-related brain potentials. *J. Psycholinguist. Res.* 30 251–266. 10.1023/A:1010490917575 11523274

[B49] Hahne A. Eckstein K. Friederici A. D. (2004). Brain signatures of syntactic and semantic processes during children’s language development. *J. Cogn. Neurosci.* 16 1302–1318. 10.1162/0898929041920504 15453981

[B50] Hahne A. Friederici A. D. (1999). Electrophysiological evidence for two steps in syntactic analysis: early automatic and late controlled processes. *J. Cogn. Neurosci.* 11 193–204. 10.1162/089892999563328 10198134

[B51] Hahne A. Friederici A. D. (2001). Processing a second language: late learners’ comprehension mechanisms as revealed by event-related brain potentials. *Biling. Lang. Cogn.* 4 123–141. 10.1017/S1366728901000232

[B52] Hahne A. Friederici A. D. (2002). Differential task effects on semantic and syntactic processes as revealed by ERPs. *Cogn. Brain Res.* 13 339–356. 10.1016/S0926-6410(01)00127-6 11918999

[B53] Hahne A. Mueller J. L. Clahsen H. (2006). Morphological processing in a second language: behavioral and event-related brain potential evidence for storage and decomposition. *J. Cogn. Neurosci.* 18 121–134. 10.1162/089892906775250067 16417688

[B54] Hatch E. Wagner-Gough J. (1976). Explaining sequence and variation in second language acquisition. *Lang. Learn.* 4 39–47.

[B55] Homae F. (2014). A brain of two halves: insights into interhemispheric organization provided by near-infrared spectroscopy. *Neuroimage* 85 354–362. 10.1016/j.neuroimage.2013.06.023 23770412

[B56] Horovitz S. G. Gore J. C. (2004). Simultaneous event-related potential and near-infrared spectroscopic studies of semantic processing. *Hum. Brain Mapp.* 22 110–115. 10.1002/hbm.20018 15108298 PMC6872128

[B57] Hoshi Y. (2003). Functional near-infrared optical imaging: utility and limitations in human brain mapping. *Psychophysiology* 40 511–520. 10.1111/1469-8986.00053 14570159

[B58] Hoshi Y. Kobayashi N. Tamura M. (2001). Interpretation of near-infrared spectroscopy signals: a study with a newly developed perfused rat brain model. *J. Appl. Physiol.* 90 1657–1662. 10.1152/jappl.2001.90.5.1657 11299252

[B59] Huttenlocher J. Haight W. Bryk A. Seltzer M. Lyons T. (1991). Early vocabulary growth: relation to language input and gender. *Dev. Psychol.* 27 236–248. 10.1037/0012-1649.27.2.236 20719872

[B60] Jang K. E. Tak S. Jung J. Jang J. Jeong Y. Ye J. C. (2009). Wavelet minimum description length detrending for near-infrared spectroscopy. *J. Biomed. Opt.* 14 1–13. 10.1117/1.3127204 19566297

[B61] Jasper H. H. (1958). The ten twenty electrode system of the international federation. *Electroencephalogr. Clin. Neurophysiol. Suppl.* 10 371–375. 10590970

[B62] Jurcak V. Okamoto M. Singh A. Dan I. (2005). Virtual 10-20 measurement on MR images for inter-modal linking of transcranial and tomographic neuroimaging methods. *Neuroimage* 26 1184–1192. 10.1016/j.neuroimage.2005.03.021 15961052

[B63] Jurcak V. Tsuzuki D. Dan I. (2007). 10/20 10/10 and 10/5 systems revisited: their validity as relative head-surface-based positioning systems. *Neuroimage* 34 1600–1611. 10.1016/j.neuroimage.2006.09.024 17207640

[B64] Just M. A. Carpenter P. A. Keller T. A. Eddy W. F. Thulborn K. R. (1996). Brain activation modulated by sentence comprehension. *Science* 274 114–116. 10.1126/science.274.5284.114 8810246

[B65] Kansaku K. Yamaura A. Kitazawa S. (2000). Sex differences in lateralization revealed in the posterior language areas. *Cereb. Cortex* 10 866–872. 10.1093/cercor/10.9.866 10982747

[B66] King J. Just M. A. (1991). Individual differences in syntactic processing: the role of working memory. *J. Mem. Lang.* 30 580–602. 10.1016/0749-596X(91)90027-H

[B67] Kotz S. A. (2009). A critical review of ERP and fMRI evidence on L2 syntactic processing. *Brain Lang.* 109 68–74. 10.1016/j.bandl.2008.06.002 18657314

[B68] Kovelman I. Shalinsky M. H. Berens M. S. Petitto L. A. (2008). Shining new light on the brain’s “bilingual signature”: a functional near infrared spectroscopy investigation of semantic processing. *Neuroimage* 39 1457–1471. 10.1016/j.neuroimage.2007.10.017 18054251 PMC2249758

[B69] Kramer J. H. Delis D. C. Kaplan E. O’donnell L. Prifitera A. (1997). Developmental sex differences in verbal learning. *Neuropsychology* 11 577–584. 10.1037/0894-4105.11.4.577 9345701

[B70] Kuperberg G. R. McGuire P. K. Bullmore E. T. Brammer M. J. Rabe-Hesketh S. Wright I. C. (2000). Common and distinct neural substrates for pragmatic, semantic and syntactic processing of spoken sentences: an FMRI study. *J. Cogn. Neurosci.* 12 321–341. 10.1162/089892900562138 10771415

[B71] Kutas M. Federmeier K. D. (2000). Electrophysiology reveals semantic memory use in language comprehension. *Trends Cogn. Sci.* 4 463–470. 10.1016/S1364-6613(00)01560-6 11115760

[B72] Kutas M. Hillyard S. A. (1980). Reading senseless sentences: brain potentials reflect semantic incongruity. *Science* 207 203–205. 10.1126/science.7350657 7350657

[B73] Lancaster J. L. Woldorff M. G. Parsons L. M. Liotti M. Freitas C. S. Rainey L. (2000). Automated Talairach atlas labels for functional brain mapping. *Hum. Brain Mapp.* 10 120–131. 10.1002/1097-0193(200007)10:3<120::AID-HBM30>3.0.CO;2-8 10912591 PMC6871915

[B74] Lehto J. (1995). Working memory and school achievement in the ninth form. *Educ. Psychol.* 15 271–281. 10.1080/0144341950150304

[B75] Lum J. A. Conti-Ramsden G. Page D. Ullman M. T. (2012). Working, declarative and procedural memory in specific language impairment. *Cortex* 48 1138–1154. 10.1016/j.cortex.2011.06.001 21774923 PMC3664921

[B76] Maki A. Yamashita Y. Ito Y. Watanabe E. Mayanagi Y. Koizumi H. (1995). Spatial and temporal analysis of human motor activity using noninvasive NIR topography. *Med. Phys.* 22 1997–2005. 10.1118/1.597496 8746704

[B77] Minagawa-Kawai Y. Cristià A. Dupoux E. (2011). Cerebral lateralization and early speech acquisition: a developmental scenario. *Dev. Cogn. Neurosci.* 1 217–232. 10.1016/j.dcn.2011.03.005 22436509 PMC6987554

[B78] Miyake A. Friedman N. P. (1998). “Individual differences in second language proficiency: working memory as language aptitude,” in *Foreign Language Learning Psycholinguistic Studies on Training and Retention*, eds Healy A. F. Bourne L. E. (Mahwah, NJ: Lawrence Erlbaum Associates), 339–364.

[B79] Mueller J. L. (2005). Electrophysiological correlates of second language processing. *Second Lang. Res.* 21 152–174. 10.1191/0267658305sr256oa

[B80] Murray A. D. Johnson J. Peters J. (1990). Fine-tuning of utterance length to preverbal infants: effects on later language development. *J. Child Lang.* 17 511–525. 10.1017/S0305000900010862 2269697

[B81] Murtha S. Chertkow H. Beauregard M. Evans A. (1999). The neural substrate of picture naming. *J. Cogn. Neurosci.* 11 399–423. 10.1162/089892999563508 10471848

[B82] Nagel B. Ohannissien A. Cummins K. (2007). Performance dissociation during verbal and spatial working memory tasks. *Percept. Motor Skill* 105 243–250. 10.2466/pms.105.1.243-250 17918571

[B83] Nelson K. (1973). Structure and strategy in learning to talk. *Monogr. Soc. Res. Child Dev.* 38 10.2307/1165788

[B84] Ni W. Constable R. T. Mencl W. E. Pugh K. R. Fulbright R. K. Shaywitz S. E. (2000). An event-related neuroimaging study distinguishing form and content in sentence processing. *J. Cogn. Neurosci.* 12 120–133. 10.1162/08989290051137648 10769310

[B85] Oi M. Saito H. Ito H. Rumme P. L. (2010). Semantic judgment of Chinese-Japanese bilinguals: a near-infrared spectroscopy study. *Neuroreport* 21 127–131. 10.1097/WNR.0b013e328334f235 19997036

[B86] Okada E. Firbank M. Schweiger M. Arridge S. R. Cope M. Delpy D. T. (1997). Theoretical and experimental investigation of near-infrared light propagation in a model of the adult head. *Appl. Opt.* 36 21–31. 10.1364/AO.36.000021 18250644

[B87] Oldfield R. C. (1971). The assessment and analysis of handedness: the Edinburgh inventory. *Neuropsychologia* 9 97–113. 10.1016/0028-3932(71)90067-4 5146491

[B88] Osaka M. Osaka N. (1992). Language-independent working memory as measured by Japanese and English reading span tests. *Bull. Psychon. Soc.* 30 287–289. 10.3758/BF03330466

[B89] Pauls F. Petermann F. Lepach A. C. (2013). Gender differences in episodic memory and visual working memory including the effects of age. *Memory* 21 857–874. 10.1080/09658211.2013.765892 23383629

[B90] Paus T. (2010). Growth of white matter in the adolescent brain: myelin or axon? *Brain Cogn.* 72 26–35. 10.1016/j.bandc.2009.06.002 19595493

[B91] Phillips M. D. Lowe M. J. Lurito J. T. Dzemidzic M. Mathews V. P. (2001). Temporal lobe activation demonstrates sex-based differences during passive listening. *Radiology* 220 202–207. 10.1148/radiology.220.1.r01jl34202 11425998

[B92] Plante E. Schmithorst V. J. Holland S. K. Byars A. W. (2006). Sex differences in the activation of language cortex during childhood. *Neuropsychologia* 44 1210–1221. 10.1016/j.neuropsychologia.2005.08.016 16303148

[B93] Plichta M. M. Herrmann M. J. Baehne C. G. Ehlis A. C. Richter M. M. Pauli P. (2006). Event-related functional near-infrared spectroscopy (fNIRS): are the measurements reliable? *Neuroimage* 31 116–124. 10.1016/j.neuroimage.2005.12.008 16446104

[B94] Pugh K. R. Shaywitz B. A. Shaywitz S. E. Constable R. T. Skudlarski P. Fulbright R. K. (1996). Cerebral organization of component processes in reading. *Brain* 119 1221–1238. 10.1093/brain/119.4.1221 8813285

[B95] Quaresima V. Bisconti S. Ferrari M. (2012). A brief review on the use of functional near-infrared spectroscopy (fNIRS) for language imaging studies in human newborns and adults. *Brain Lang.* 121 79–89. 10.1016/j.bandl.2011.03.009 21507474

[B96] Rossi S. Gugler M. F. Friederici A. D. Hahne A. (2006). The impact of proficiency on syntactic second-language processing of German and Italian: evidence from event-related potentials. *J. Cogn. Neurosci.* 18 2030–2048. 10.1162/jocn.2006.18.12.2030 17129189

[B97] Rossi S. Gugler M. F. Hahne A. Friederici A. D. (2005). When word category information encounters morphosyntax: an ERP study. *Neurosci. Lett.* 384 228–233. 10.1016/j.neulet.2005.04.077 15893877

[B98] Roulstone S. Loader S. Northstone K. Beveridge M. (2002). The speech and language of children aged 25 months: descriptive data from the Avon longitudinal study of parents and children. *Early Child Dev. Care* 172 259–268. 10.1080/03004430212126

[B99] Rüschemeyer S. A. Zysset S. Friederici A. D. (2006). Native and nonnative reading of sentences: an fMRI experiment. *Neuroimage* 31 354–365. 10.1016/j.neuroimage.2005.11.047 16427323

[B100] Sambataro F. Reed J. D. Murty V. P. Das S. Tan H. Y. Callicott J. H. (2009). Catechol-*O*-methyltransferase valine^158^methionine polymorphism modulates brain networks underlying working memory across adulthood. *Biol. Psychiatry* 66 540–548. 10.1016/j.biopsych.2009.04.014 19539269 PMC3085346

[B101] Sankoh A. J. Huque M. F. Dubey S. D. (1997). Some comments on frequently used multiple endpoint adjustment methods in clinical trials. *Stat. Med.* 16 2529–2542. 10.1002/(SICI)1097-0258(19971130)16:22<2529::AID-SIM692>3.0.CO;2-J 9403954

[B102] Sassaroli A. Tong Y. Benes C. Fantini S. (2008). Data analysis and statistical tests for near-infrared functional studies of the brain. *Proc. SPIE* 6850:685008 10.1117/12.761707

[B103] Schecklmann M. Giani A. Tupak S. Langguth B. Raab V. (2014). Functional near-infrared spectroscopy to probe state- and trait-like conditions in chronic tinnitus: a proof-of-principle study. *Neural Plast.* 2014:894203. 10.1155/2014/894203 25478237 PMC4248328

[B104] Semlitsch H. V. Anderer P. Schuster P. Presslich O. (1986). A solution for reliable and valid reduction of ocular artifacts applied to the P300 ERP. *Psychophysiology* 23 695–703. 10.1111/j.1469-8986.1986.tb00696.x 3823345

[B105] Shattuck D. W. Mirza M. Adisetiyo V. Hojatkashani C. Salamon G. Narr K. L. (2008). Construction of a 3D probabilistic atlas of human cortical structures. *Neuroimage* 39 1064–1080. 10.1016/j.neuroimage.2007.09.031 18037310 PMC2757616

[B106] Shaywitz B. A. Shaywitz S. E. Pugh K. R. Constable R. T. Skudlarski P. Fulbright R. K. (1995). Sex differences in the functional organization of the brain for language. *Nature* 373 607–609. 10.1038/373607a0 7854416

[B107] Singh A. K. Okamoto M. Dan H. Jurcak V. Dan I. (2005). Spatial registration of multi-channel multi-subject fNIRS data to MNI space without MRI. *Neuroimage* 27 842–851. 10.1016/j.neuroimage.2005.05.019 15979346

[B108] Sisk C. L. Zehr J. L. (2005). Pubertal hormones organize the adolescent brain and behavior. *Front. Neuroendocrinol.* 26 163–174. 10.1016/j.yfrne.2005.10.00 16309736

[B109] Sommer I. E. Aleman A. Bouma A. Kahn R. S. (2004). Do women really have more bilateral language representation than men? A meta-analysis of functional imaging studies. *Brain* 127 1845–1852. 10.1093/brain/awh207 15240433

[B110] Sommer I. E. Aleman A. Somers M. Boks M. P. Kahn R. S. (2008). Sex differences in handedness, asymmetry of the planum temporale and functional language lateralization. *Brain Res.* 1206 76–88. 10.1016/j.brainres.2008.01.003 18359009

[B111] Speck O. Ernst T. Braun J. Koch C. Miller E. Chang L. (2000). Gender differences in the functional organization of the brain for working memory. *Neuroreport* 11 2581–2585. 10.1097/00001756-200008030-00046 10943726

[B112] Stokes P. R. Rhodes R. A. Grasby P. M. Mehta M. A. (2011). The effects of the COMT Val^108^/^158^Met polymorphism on BOLD activation during working memory, planning, and response inhibition: a role for the posterior cingulate cortex? *Neuropsychopharmacology* 36 763–771. 10.1038/npp.2010.210 21150912 PMC3055733

[B113] Strangman G. Culver J. P. Thompson J. H. Boas D. A. (2002). A quantitative comparison of simultaneous BOLD fMRI and NIRS recordings during functional brain activation. *Neuroimage* 17 719–731. 10.1006/nimg.2002.1227 12377147

[B114] Stromswold K. Caplan D. Alpert N. Rauch S. (1996). Localization of syntactic comprehension by positron emission tomography. *Brain Lang.* 52 452–473. 10.1006/brln.1996.0024 8653390

[B115] Sugiura L. Ojima S. Matsuba-Kurita H. Dan I. Tsuzuki D. Katura T. (2011). Sound to language: different cortical processing for first and second languages in elementary school children as revealed by a large-scale study using fNIRS. *Cereb. Cortex* 10 2374–2393. 10.1093/cercor/bhr023 21350046 PMC3169662

[B116] Sugiura L. Ojima S. Matsuba-Kurita H. Dan I. Tsuzuki D. Katura T. (2015). Effects of sex and proficiency in second language processing as revealed by a large-scale fNIRS study of school-aged children. *Hum. Brain Mapp.* 36 3890–3911. 10.1002/hbm.22885 26147179 PMC6868995

[B117] Sugiura L. Toyota T. Matsuba-Kurita H. Iwayama Y. Mazuka R. Yoshikawa T. (2017). Age-dependent effects of catechol-*O*-methyltransferase (*COMT*) gene Val^158^Met polymorphism on language function in developing children. *Cereb. Cortex* 27 104–116. 10.1093/cercor/bhw371 27909011 PMC6044402

[B118] Suh S. Yoon H. W. Lee S. Chung J. Y. Cho Z. H. Park H. W. (2007). Effects of syntactic complexity in L1 and L2: an fMRI study of Korean–English bilinguals. *Brain Res.* 1136 178–189. 10.1016/j.brainres.2006.12.043 17229404

[B119] Tak S. Ye J. C. (2014). Statistical analysis of fNIRS data: a comprehensive review. *Neuroiimage* 85(Pt 1), 72–91. 10.1016/j.neuroimage.2013.06.016 23774396

[B120] Tan H. Y. Chen Q. Goldberg T. E. Mattay V. S. Meyer-Lindenberg A. Weinberger D. R. (2007). Catechol-O-methyltransferase Val158Met modulation of prefrontal-parietal-striatal brain systems during arithmetic and temporal transformations in working memory. *J. Neurosci.* 27 13393–13401. 10.1523/JNEUROSCI.4041-07.2007 18057197 PMC6673107

[B121] Tatsuno Y. Sakai K. L. (2005). Language-related activations in the left prefrontal regions are differentially modulated by age, proficiency and task demands. *J. Neurosci.* 16 1637–1644. 10.1523/JNEUROSCI.3978-04.2005 15716399 PMC6725945

[B122] Tzourio-Mazoyer N. Landeau B. Papathanassiou D. Crivello F. Etard O. Delcroix N. (2002). Automated anatomical labeling of activations in SPM using a macroscopic anatomical parcellation of the MNI MRI single-subject brain. *Neuroimage* 15 273–289. 10.1006/nimg.2001.0978 11771995

[B123] Uga M. Dan I. Sano T. Dan H. Watanabe E. (2014). Optimizing the general linear model for functional near-infrared spectroscopy: an adaptive hemodynamic response function approach. *Neurophoton* 1:015004. 10.1117/1.NPh.1.1.015004 26157973 PMC4478847

[B124] Ullman M. T. (2001). A neurocognitive perspective on language: the declarative/procedural model. *Nat. Rev. Neurosci.* 2 717–726. 10.1038/35094573 11584309

[B125] Ullman M. T. Corkin S. Coppola M. Hickok G. Growdon J. H. Koroshetz W. J. (1997). A neural dissociation within language: evidence that the mental dictionary is part of declarative memory, and that grammatical rules are processed by the procedural system. *J. Cogn. Neurosci.* 9 266–276. 10.1162/jocn.1997.9.2.266 23962016

[B126] Vandenberghe R. Price C. Wise R. Josephs O. Frackowiak R. S. (1996). Functional anatomy of a common semantic system for words and pictures. *Nature* 383 254–256. 10.1038/383254a0 8805700

[B127] Vannest J. J. Karunanayaka P. R. Altaye M. Schmithorst V. J. Plante E. M. Eaton K. J. (2009). Comparison of fMRI data from passive listening and active-response story processing tasks in children. *J. Magn. Reson. Imaging* 29 971–976. 10.1002/jmri.21694 19306445 PMC2763568

[B128] Vigneau M. Beaucousin V. Hervé P. Y. Duffau H. Crivello F. Houdé O. (2006). Meta-analyzing left hemisphere language areas: phonology, semantics, and sentence processing. *Neuroimage* 30 1414–1432. 10.1016/j.neuroimage.2005.11.002 16413796

[B129] Vijayraghavan S. Wang M. Birnbaum S. G. Williams G. V. Arnsten A. F. (2007). Inverted-U dopamine D1 receptor actions on prefrontal neurons engaged in working memory. *Nat. Neurosci.* 10 376–384. 10.1038/nn1846 17277774

[B130] Wahlstrom D. White T. Hooper C. J. Vrshek-Schallhorn S. Oetting W. S. Brott M. J. (2007). Variations in the catechol *O*-methyltransferase polymorphism and prefrontally guided behaviors in adolescents. *Biol. Psychiatry* 61 626–632. 10.1016/j.biopsych.2006.05.045 17014828

[B131] Wallentin M. (2009). Putative sex differences in verbal abilities and language cortex: a critical review. *Brain Lang.* 108 175–183. 10.1016/j.bandl.2008.07.001 18722007

[B132] Wallois F. Mahmoudzadeh M. Patil A. Grebe R. (2012). Usefulness of simultaneous EEG-NIRS recording in language studies. *Brain Lang.* 121 110–123. 10.1016/j.bandl.2011.03.010 21546072

[B133] Weber K. Lavric A. (2008). Syntactic anomaly elicits a lexico-semantic (N400) ERP effect in the second language but not the first. *Psychophysiology* 45 920–925. 10.1111/j.1469-8986.2008.00691.x 18778320

[B134] Weiss E. M. Siedentopf C. Hofer A. Deisenhammer E. A. Hoptman M. J. Kremser C. (2003). Brain activation pattern during a verbal fluency test in healthy male and female volunteers: a functional magnetic resonance imaging study. *Neurosci. Lett.* 352 191–194. 10.1016/j.neulet.2003.08.071 14625017

[B135] Wiggs C. L. Weisberg J. Martin A. (1999). Neural correlates of semantic and episodic memory retrieval. *Neuropsychologia* 37 103–118. 10.1016/S0028-3932(98)00044-X 9920476

